# Microbiota-Derived Metabolites in the Epigenetic Regulation of Redox Homeostasis

**DOI:** 10.3390/antiox15070897

**Published:** 2026-07-20

**Authors:** Patricia Mester, Sara Martina Steinmann, Simon Mehler, Martina Müller, Karsten Gülow

**Affiliations:** Department of Internal Medicine I, Gastroenterology, Hepatology, Endocrinology, Rheumatology, Immunology, and Infectious Diseases, University Hospital Regensburg, 93053 Regensburg, Germany; patricia.mester-pavel@klinik.uni-regensburg.de (P.M.); sara.steinmann@klinik.uni-regensburg.de (S.M.S.); simon.mehler@stud.uni-regensburg.de (S.M.); martina.mueller-schilling@ukr.de (M.M.)

**Keywords:** epigenetic modification, methylation, acetylation, microbiota, metabolites, intestinal barrier

## Abstract

Redox homeostasis is essential for intestinal and systemic health and is regulated by antioxidant defense systems and redox-sensitive signaling pathways such as the nuclear factor erythroid 2-related factor 2 (Nrf2) and the nuclear factor ‘kappa-light-chain-enhancer’ of activated B-cells (NF-κB). Disturbances in this balance promote oxidative stress, chronic inflammation, and disease progression. Increasing evidence indicates that microbiota-derived metabolites act as key modulators of redox biology by shaping host gene expression through receptor-mediated signaling, metabolic regulation, and chromatin-associated mechanisms, including histone modifications, DNA methylation, and changes in chromatin accessibility. This review discusses how major classes of microbiota-derived and microbiota-modulated metabolites, including short-chain fatty acids (SCFAs), secondary bile acids, tryptophan-derived metabolites, polyphenol metabolites, hydrogen sulfide, and lipid mediators, influence redox-sensitive signaling and epigenetic regulation. We highlight their effects on intestinal barrier integrity and immune cell function, with particular emphasis on macrophage polarization and T-cell differentiation. Finally, we consider the emerging translational relevance of the microbiota–metabolite–epigenetic axis, while emphasizing that biomarker development and therapeutic applications require further mechanistic validation and clinical studies.

## 1. Introduction

The human gastrointestinal tract harbors a highly complex and dynamic microbial ecosystem that plays a fundamental role in maintaining host homeostasis. Beyond its established functions in digestion and nutrient processing, the gut microbiota contributes to immune regulation, epithelial barrier integrity, and systemic metabolic control. Alterations in the composition, diversity, and function of this microbial community, commonly referred to as dysbiosis, have been associated with a broad spectrum of disorders, including inflammatory, metabolic, and neurodegenerative diseases. These disease associations are thought to arise, at least in part, from impaired barrier function, dysregulated immune signaling, and changes in the production of bioactive microbial metabolites [1,2,3,4,5].

The maintenance of redox homeostasis is essential for cellular function and cellular, tissue, and organismal homeostasis. Reactive oxygen species (ROS) are continuously generated as by-products of cellular metabolism and serve important signaling functions under physiological conditions [6]. However, excessive ROS production or impaired antioxidant defense systems result in oxidative stress, which contributes to chronic inflammation, cellular damage, and the development of a wide range of diseases [6]. In the intestinal environment, where host tissues are in constant contact with microbial signals and metabolic products, tight regulation of redox balance is particularly critical [7]. Disruption of this balance has been implicated in inflammatory disorders, metabolic dysfunction, and neurodegenerative and age-related diseases, highlighting oxidative stress as a central mediator linking environmental and microbial factors to disease pathogenesis [8,9,10,11].

A central mechanism by which the gut microbiota influences host physiology is the production of bioactive metabolites that mediate host–microbiota interactions. Among these, short-chain fatty acids (SCFAs), secondary bile acids, and tryptophan-derived metabolites have emerged as key signaling molecules that modulate immune responses, cellular metabolism, and stress adaptation pathways [4,12,13,14]. These metabolites act through diverse mechanisms, including receptor-mediated signaling, modulation of intracellular metabolic pathways, and transcriptional and chromatin-associated regulatory mechanisms. Importantly, growing evidence indicates that microbial metabolites can also influence host responses at the epigenetic level by affecting histone acetylation, chromatin accessibility, and other transcription-associated regulatory processes [5,15].

In this way, epigenetic regulation may represent a mechanistic interface through which microbiota-derived metabolites shape redox-sensitive signaling pathways and antioxidant defense programs. SCFAs have been shown to inhibit histone deacetylases (HDACs) and modulate chromatin accessibility, thereby linking microbial metabolism to transcriptional control [13,16,17]. Similarly, other metabolite classes, including bile acid derivatives and tryptophan metabolites, can affect transcriptional programs through ligand-activated transcription factor signaling, nuclear receptor signaling, and chromatin-associated regulatory mechanisms [4,14]. Despite these advances, the role of microbiota-derived metabolites as modulators of epigenetic and chromatin-associated regulation of host redox homeostasis remains incompletely integrated across metabolite classes, epigenetic mechanisms, and redox-sensitive signaling pathways. Although previous work has highlighted the interplay between gut microbiota, oxidative stress, and immune regulation [18], the integration of microbial metabolism, epigenetic control, and redox signaling into a coherent mechanistic framework remains limited. This review, therefore, brings together evidence from different metabolite classes and discusses how these microbial signals converge on chromatin-associated regulation, redox-sensitive signaling, epithelial barrier function, and immune regulation. To address this gap, this narrative review focuses on mechanistic and conceptual evidence linking microbiota-derived and microbiota-modulated metabolites to epigenetic regulation and redox homeostasis. Relevant literature was identified through targeted searches of PubMed, Web of Science, and Google Scholar using combinations of terms related to gut microbiota, microbial metabolites, epigenetic regulation, chromatin accessibility, oxidative stress, redox homeostasis, Nuclear factor erythroid 2-related factor 2 (Nrf2), nuclear factor ‘kappa-light-chain-enhancer’ of activated B-cells (NF-κB), aryl hydrocarbon receptor (AhR), intestinal barrier function, inflammation, and immune regulation. Publications with emphasis on original research articles, mechanistic studies, and relevant reviews were considered. The structure of the review follows the main metabolite classes and highlights how they intersect with epigenetic regulation, redox-sensitive signaling, and biological function. The guiding question of this review is how microbiota-derived and microbiota-modulated metabolites influence redox homeostasis through epigenetic and chromatin-associated mechanisms, while distinguishing mechanistically supported pathways from context-dependent or still emerging concepts.

## 2. Microbiota-Derived Metabolites: The Key Players

The microbial community of the distal gut is vast and diverse and forms a dynamic ecosystem in close association with its host. Interactions among microbial taxa, as well as between microbial communities and the host, play a crucial role in shaping host physiology [19]. The microbiota contributes to protection against enteric infections [20,21,22], supports the development and regulation of the immune system and intestinal epithelium [23], and contributes to nutrient metabolism, energy harvest, and metabolic homeostasis [24,25,26]. Disruptions of this balanced community can result in an altered state referred to as dysbiosis. Host–microbiota interactions are partly mediated by small-molecule metabolites, which act through diverse mechanisms, including as receptor ligands, enzyme inhibitors, metabolic substrates, or metabolic precursors [27,28]. This microbial metabolome is shaped by both the composition of the microbiota and the host’s diet [26,29,30].

Among microbiota-derived metabolites, SCFAs, such as acetate, propionate, and butyrate, are of particular importance. These metabolites are generated by bacterial fermentation of dietary fiber in the colon and represent central mediators of diet–microbiota–host interactions [12,13,31,32]. As the most abundant and best-characterized microbiota-derived metabolites, SCFAs provide an important conceptual framework for understanding how microbial metabolites influence host physiology, including redox-associated and epigenetically regulated processes [13,33,34,35,36]. However, SCFAs should be viewed as one major component of a broader metabolite network that also includes secondary bile acids, tryptophan-derived metabolites, polyphenol-derived metabolites, hydrogen sulfide, and lipid mediators, all of which can contribute to host signaling, immune regulation, and redox homeostasis in a context-dependent manner [4,28]. Importantly, these metabolites rarely act in isolation. Their biological effects are shaped by the surrounding metabolite milieu, the responding cell type, and the inflammatory or metabolic state of the tissue, which may explain why findings from simplified experimental models are not always directly comparable.

### 2.1. Short-Chain Fatty Acids (SCFAs)

SCFAs, particularly acetate, propionate, and butyrate, are key microbiota-derived metabolites generated through the anaerobic fermentation of dietary fibers in the colon [7,32]. They reach millimolar concentrations in the gut lumen and, to a lesser extent, in systemic circulation, where they exert pleiotropic effects on host metabolism, immune regulation, and cellular homeostasis [7,31,37]. SCFAs contribute to immune homeostasis, influence energy metabolism, regulate mucosal immunity, shape T-cell polarization and plasticity, and affect B-cell responses and antibody production [38,39].

**Figure 1:**
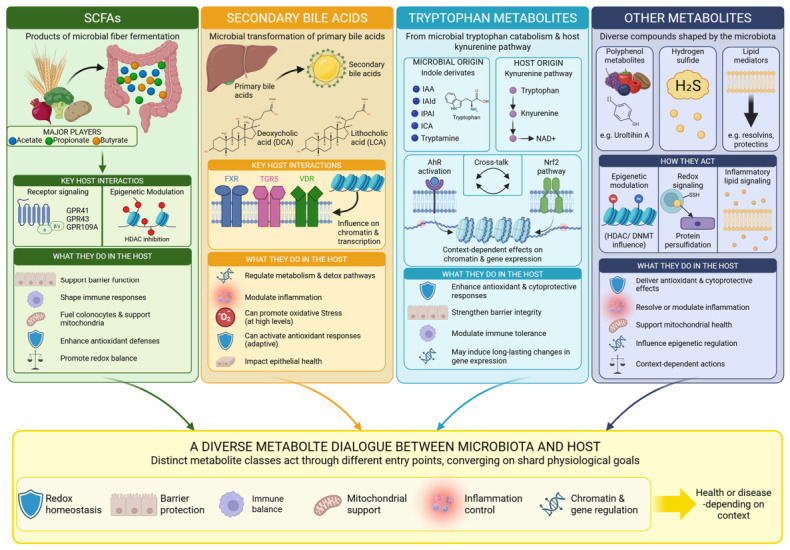
Major classes of microbiota-derived metabolites and their principal routes of host interaction. Microbiota-derived and microbiota-modulated metabolites comprise several major functional classes, including SCFAs, secondary bile acids, tryptophan metabolites, and other bioactive compounds such as polyphenol-derived metabolites, H_2_S, and lipid mediators. Although these metabolite groups differ in origin, chemical structure, and primary modes of action, they converge on shared host processes through receptor-mediated signaling, epigenetic and chromatin-associated regulation, redox signaling, and immunometabolic pathways. Collectively, these interactions influence barrier integrity, immune homeostasis, mitochondrial function, inflammatory control, and redox balance, thereby contributing to either tissue protection and homeostasis or, in a context-dependent manner, disease-promoting responses. Abbreviations: H_2_S, hydrogen sulfide; SCFAs, short-chain fatty acids. Created in Biorender. https://BioRender.com (accessed on 29 May 2026).

A central mechanism by which SCFAs influence host physiology is through epigenetic regulation. Butyrate and, to a lesser extent, propionate act as inhibitors of HDACs, leading to increased histone acetylation and transcriptional activation of target genes [16,40,41]. Acetate is generally considered a weaker HDAC inhibitor but can contribute to histone acetylation indirectly by serving as a precursor for acetyl-CoA generation, depending on cellular context. In addition, SCFAs may affect acetyl-CoA-dependent chromatin regulation, as butyrate has also been shown to promote p300 acetyltransferase activity, further linking microbial metabolism to host chromatin remodeling and gene expression (Figure 1) [17,42].

In intestinal epithelial cells and immune cells, SCFA-mediated HDAC inhibition promotes the expression of genes involved in barrier function, anti-inflammatory responses, and cellular stress resistance. For example, butyrate enhances the differentiation of regulatory T cells (Tregs) through epigenetic upregulation of FOXP3 expression [40,43]. Beyond chromatin remodeling, butyrate can also support induced Treg differentiation through metabolic reprogramming, including enhanced fatty acid oxidation [44]. Similarly, SCFAs have been shown to regulate histone acetylation at promoters of antioxidant genes, thereby modulating cellular redox responses. However, the extent to which these antioxidant effects are directly epigenetically mediated versus secondary to metabolic or receptor-dependent signaling remains cell type- and context-dependent.

Beyond histone acetylation, emerging evidence suggests that SCFAs may also influence other epigenetic modifications, including DNA methylation and histone crotonylation. These effects are less extensively characterized than HDAC inhibition and should therefore be interpreted as emerging rather than established mechanisms. Notably, butyrate-derived crotonyl-CoA has been implicated in histone crotonylation, a modification associated with active gene transcription [45].

SCFAs play a crucial role in maintaining redox homeostasis by modulating both the production of ROS and the cellular antioxidant defense systems. Butyrate has been shown to activate the Nrf2 pathway, a master regulator of antioxidant responses, leading to the upregulation of genes such as heme oxygenase 1 (HO-1), NAD(P)H quinone dehydrogenase 1 (NQO1), and genes involved in glutathione synthesis and detoxification, including glutamate–cysteine ligase catalytic subunit (GCLC)/Glutamate-cysteine ligase regulatory subunit (GCLM) [7,46,47]. Given the central role of Nrf2 in coordinating antioxidant and metabolic responses, this pathway provides an important mechanistic interface between microbial metabolites and redox homeostasis [8]. Mechanistically, this effect is partly mediated through epigenetic modulation, as HDAC inhibition facilitates chromatin accessibility at antioxidant response elements (AREs), enhancing Nrf2 target gene transcription. In parallel, SCFAs can suppress pro-oxidant pathways, including NF-κB signaling, thereby reducing inflammation-associated oxidative stress [48]. In mitochondria, SCFAs—particularly butyrate—serve as an energy substrate for colonocytes and improve mitochondrial function and biogenesis. This contributes indirectly to redox balance by reducing electron leakage and can support epithelial oxygen consumption and mitochondrial metabolism, thereby contributing indirectly to redox balance [49,50,51]. However, mitochondrial effects are context-dependent: while butyrate supports oxidative metabolism in differentiated colonocytes, it may exert different or even growth-inhibitory effects in transformed cells or under inflammatory conditions. However, the effects of SCFAs on oxidative stress appear to be context-dependent, influenced by concentration, cell type, and disease state. Under certain conditions, butyrate may also promote the expression of pro-inflammatory or T-cell subset-specific programs [52,53].

In addition to their intracellular epigenetic effects, SCFAs signal through G-protein-coupled receptors (GPCRs), including GPR41 (FFAR3), GPR43 (FFAR2), and GPR109A. Activation of these receptors modulates immune and metabolic pathways that intersect with redox regulation. For instance, GPR109A activation by butyrate has been linked to anti-inflammatory signaling and protection against oxidative damage in colonic tissues [54]. The recognition of SCFAs by GPR41 and GPR43 further supports the view that these metabolites act not only as metabolic intermediates but also as signaling molecules with broad immunoregulatory effects [55,56,57]. Importantly, receptor-mediated signaling and epigenetic modulation are not mutually exclusive but rather complementary. SCFAs can simultaneously activate GPCRs and inhibit HDACs, resulting in coordinated regulation of gene expression and cellular responses to oxidative stress.

SCFAs represent a critical link between the gut microbiota and host epigenetic regulation of redox homeostasis. Through HDAC inhibition, modulation of histone modifications, metabolic effects on acetyl-CoA and mitochondrial function, and activation of signaling pathways such as Nrf2 and GPCR-mediated cascades, SCFAs orchestrate a complex network that integrates metabolic, immune, and oxidative stress responses. These mechanisms illustrate how diet–microbiota interactions can shape redox-associated gene regulation and may provide entry points for therapeutic intervention.

### 2.2. Secondary Bile Acids

Secondary bile acids (SBAs), including deoxycholic acid (DCA) and lithocholic acid (LCA), are microbiota-derived metabolites generated from primary bile acids through bacterial deconjugation and enzymatic transformations such as 7α-dehydroxylation, dehydrogenation, and epimerization in the intestine (Figure 1). These transformations are carried out by specialized members of the gut microbiota, including several anaerobic taxa within the Firmicutes, particularly Clostridium cluster XIVa species, linking microbial composition directly to the host bile acid pool and its downstream signaling effects [58,59]. Beyond their classical role in lipid absorption, SBAs have emerged as modulators of host signaling and transcriptional regulation. Several SBAs have been reported to influence chromatin-associated processes. However, compared with SCFAs, the evidence that SBAs act as direct epigenetic enzyme modulators is less extensive and is often indirect, receptor-mediated, or disease-context dependent. LCA, DCA, and their derivatives have been shown to influence gene expression programs related to metabolism, inflammation, detoxification, and stress responses, partly through modulation of bile acid-responsive transcription factors and signaling pathways. Some studies suggest that bile acids can affect histone acetylation, DNA methylation, or chromatin accessibility, but these effects should be interpreted primarily as chromatin-associated consequences of bile acid signaling or chronic exposure rather than as uniformly established direct HDAC or histone acetyltransferase (HAT) inhibition [60,61,62]. This distinction is important because many reported epigenetic changes occur in settings of prolonged or high-level bile acid exposure, particularly in inflammatory or neoplastic disease models. Therefore, such findings may reflect adaptive or pathological remodeling of the cellular state rather than a direct and generalizable epigenetic action of secondary bile acids.

In addition, bile acid signaling through nuclear receptors such as the farnesoid X receptor (FXR) and the vitamin D receptor (VDR) has important transcriptional and epigenetic consequences. Activation of these receptors leads to recruitment of chromatin-modifying complexes, thereby altering gene expression programs related to inflammation, detoxification, and oxidative stress [63,64]. Beyond these receptor-mediated effects on chromatin regulation, emerging evidence also links bile acids to DNA methylation changes, particularly in the context of chronic exposure and disease states such as colorectal cancer, where DCA has been associated with epigenetic dysregulation and aberrant gene silencing [65]. These observations suggest that bile acid-driven epigenetic regulation may occur both through ligand-activated transcription factor signaling and through more persistent changes in epigenetic state. However, such effects are often disease- and context-dependent and may reflect long-term adaptive or pathological responses rather than direct epigenetic targeting. At the functional level, these epigenetic and receptor-mediated effects are closely linked to redox homeostasis. SBAs exert complex, often context-dependent effects on redox homeostasis. On the one hand, hydrophobic bile acids such as DCA and LCA can induce oxidative stress by promoting mitochondrial dysfunction, increasing ROS production, and triggering lipid peroxidation [66,67]. These pro-oxidant effects are particularly evident at high concentrations and have been implicated in mucosal damage and carcinogenesis in the colon. On the other hand, bile acid signaling can also activate adaptive cytoprotective and antioxidant pathways. For example, FXR activation has been shown to modulate oxidative stress by regulating genes involved in glutathione metabolism and detoxification pathways. Similarly, G protein-coupled bile acid receptor 1 (TGR5/GPBAR1), a G-protein-coupled bile acid receptor, can induce anti-inflammatory and cytoprotective signaling that indirectly reduces oxidative stress [68,69,70].

Interestingly, LCA has been reported to activate the Nrf2 pathway under certain conditions, promoting the expression of antioxidant enzymes and contributing to cellular resilience. This should be presented as a context-dependent adaptive stress response rather than as a general antioxidant property of SBAs. These observations suggest that bile acids can modulate both pro-oxidant and antioxidant pathways depending on bile acid species, concentration, cell type, and exposure duration [71]. This dual behavior also helps to explain why findings across studies are not always consistent. Hydrophobic bile acids may induce oxidative stress and mitochondrial dysfunction in epithelial or transformed cells, whereas receptor-mediated FXR, VDR, or TGR5 signaling can activate compensatory cytoprotective programs in other settings. Thus, the biological outcome of SBA exposure depends strongly on whether acute signaling responses, chronic stress adaptation, or disease-associated tissue remodeling are being examined.

A key feature of SBA biology is the tight integration of receptor-mediated signaling with epigenetic regulation. FXR and VDR function as ligand-activated transcription factors that recruit co-activators or co-repressors with histone-modifying activity, thereby linking bile acid sensing directly to chromatin remodeling [72,73,74,75]. Moreover, crosstalk between SBA signaling and inflammatory pathways such as NF-κB further influences the epigenetic control of redox-related genes. For instance, bile acid-mediated repression of NF-κB activity can reduce the transcription of pro-oxidant and pro-inflammatory genes, while epigenetic modifications may contribute to the stabilization of these transcriptional changes over time [76,77,78,79]. Whether such transcriptional effects become stabilized through durable epigenetic remodeling likely depends on cell type, disease context, and duration of exposure.

The production and composition of SBAs are highly dependent on gut microbiota structure, making them a dynamic interface between diet, microbiome, and host physiology. Dysbiosis can shift the bile acid pool toward more hydrophobic and potentially cytotoxic species, thereby enhancing oxidative stress and epigenetic dysregulation [80]. This is particularly relevant because changes in SBA composition are unlikely to act in isolation, but rather within a broader metabolite environment shaped by the microbiota. Importantly, there is significant interplay between SBAs and other microbiota-derived metabolites, including SCFAs. While SCFAs generally exert anti-inflammatory and antioxidant effects through HDAC inhibition, SBAs can have both protective and deleterious roles [66]. The balance between these metabolite classes is therefore critical for maintaining redox homeostasis and epigenetic stability [81]. Secondary bile acids are key microbiota-derived signaling molecules that influence host redox homeostasis through a combination of epigenetic mechanisms and receptor-mediated pathways [66]. A better understanding of how receptor signaling, chromatin regulation, and redox effects intersect in SBA biology may provide new insights into microbiota–host interactions and identify novel therapeutic targets for oxidative stress-related diseases [82].

### 2.3. Tryptophan Metabolites

Tryptophan metabolism represents a central regulatory axis linking the gut microbiota to host cellular signaling, with emerging evidence highlighting its role in the regulation of redox homeostasis and, in selected experimental and disease contexts, chromatin-associated control of gene expression (Figure 1) [15,83]. Microbiota-derived tryptophan metabolites, particularly indole derivatives, can act as endogenous ligands or modulators of AhR, thereby influencing transcriptional programs that extend beyond classical xenobiotic responses to include immune regulation, barrier function, oxidative stress responses, and context-dependent chromatin remodeling [15,83,84,85].

Gut microbial catabolism of tryptophan generates a spectrum of indole derivatives, including indole-3-acetic acid (IAA), indole-3-aldehyde (IAld), indole-3-propionic acid (IPA), indole-3-lactic acid (ILA), and tryptamine. Not all of these metabolites activate AhR with the same potency or efficacy, and some may also signal through AhR-independent pathways such as pregnane X receptor (PXR) or other host receptors. These metabolites function as signaling molecules capable of modulating host gene expression, barrier integrity, and immune responses, in part via AhR activation [86,87]. Importantly, the biological consequences of AhR activation are highly ligand-specific and context-dependent, and different indole derivatives may induce distinct transcriptional outcomes depending on cell type, tissue environment, inflammatory state, receptor expression, and exposure duration [87,88,89]. This ligand- and context-dependency is a major reason why findings from different studies are sometimes difficult to align. Experimental systems often differ in the specific indole derivative used, its concentration, the responding cell type, and the inflammatory background, which can shift AhR signaling from barrier-protective and anti-inflammatory responses toward different or even opposing transcriptional programs.

AhR signaling has been increasingly linked to chromatin-associated regulation. Upon ligand binding, AhR translocates to the nucleus, dimerizes with aryl hydrocarbon receptor nuclear translocator (ARNT), and binds xenobiotic response elements/dioxin response elements to regulate target gene transcription. This process can involve the recruitment or displacement of chromatin-modifying co-regulators, including p300/CBP-containing HAT complexes and HDAC-containing repressor complexes [90,91]. This enables ligand-specific and context-dependent transcriptional responses. In addition, AhR has been proposed to function as a DNA methylation reader with preferential binding to unmethylated xenobiotic response elements, adding a further layer of epigenetic selectivity to AhR-dependent transcriptional control [92]. However, these findings should not be interpreted as evidence that microbial tryptophan metabolites directly modify DNA methylation; rather, they indicate that the methylation state of AhR-binding sites can influence AhR-dependent transcription. Thus, current evidence supports a close connection between tryptophan-derived metabolites, AhR-dependent transcription, and chromatin-associated regulation, but the extent to which individual microbial indoles induce stable epigenetic changes remains less clear.

Microbial tryptophan metabolites are not only signaling intermediates but also key determinants of host transcriptional landscapes, supporting the concept that sustained receptor activation may contribute to longer-lasting transcriptional reprogramming that involves chromatin-level regulation [84].

Activation of AhR by microbial tryptophan metabolites modulates oxidative stress responses through transcriptional and potentially chromatin-associated mechanisms. These metabolites attenuate oxidative stress and inflammation via AhR signaling, including suppression of pro-inflammatory cytokines, support of epithelial barrier function, and modulation of ROS-associated pathways [15]. Current evidence therefore supports a functional link between microbiota-derived tryptophan metabolites, AhR activation, inflammation, barrier integrity, and redox regulation, although the precise contribution of direct epigenetic mechanisms may vary by metabolite, model system, and tissue context [15].

At the chromatin level, AhR can recruit co-regulators such as p300/CBP, leading to histone acetylation at selected AhR-responsive loci. Additionally, AhR has been shown to interact with HDAC complexes, thereby restraining AhR-dependent transcription before ligand-induced activation or repressing selected inflammatory gene programs [91]. These chromatin-level changes provide a mechanistic basis for the sustained effects of microbial metabolites on host redox balance. However, direct evidence that microbial indole metabolites cause stable epigenetic imprinting remains limited and should be presented cautiously. Such interactions may enable ligand-specific chromatin remodeling and sustained transcriptional responses [84]. AhR activation influences T cell differentiation via transcriptional reprogramming, further supporting a role in durable changes in gene regulation [93]. More recent work has reinforced that AhR signaling can induce long-lasting changes in chromatin accessibility, consistent with sustained chromatin remodeling and possible epigenetic imprinting in selected settings [93].

A central feature of redox regulation is the interplay between AhR and the transcription factor Nrf2, a master regulator of antioxidant defense. This crosstalk is increasingly recognized as transcriptionally and chromatin-associated, but it should not be described as uniformly epigenetically controlled [94,95]. Nrf2 activity is tightly regulated by chromatin accessibility and histone modifications at ARE-containing genes. Microbial metabolites can influence this system via AhR-dependent mechanisms [48]. AhR activation may promote chromatin accessibility at Nrf2 target genes through histone acetylation, and shared coactivators such as CBP/p300 further connect both pathways at the level of transcriptional regulation [94,95]. Sustained AhR signaling may also contribute to a permissive transcriptional state for antioxidant gene induction, thereby facilitating subsequent Nrf2-mediated induction [94].

Microbial metabolites enhance gut barrier integrity via Nrf2 activation, associated with increased expression of antioxidant and tight junction genes. Although these findings are not always framed explicitly in epigenetic terms, they are compatible with chromatin-associated regulation of sustained antioxidant responses [15]. Importantly, Nrf2 target gene expression is influenced by chromatin accessibility and histone modifications such as H3K27 acetylation and H3K4 methylation. Therefore, microbiota-derived signals may shape antioxidant responses through chromatin-associated transcriptional regulation, but direct evidence should be cited specifically for each metabolite and cell type.

Beyond receptor-mediated effects, tryptophan metabolites may also affect epigenetic regulation more directly. However, this should be formulated cautiously because direct epigenetic effects of indole metabolites are less well established than AhR-mediated transcriptional effects. Kynurenine pathway metabolites regulate NAD^+^ levels and thereby modulate the activity of sirtuins such as SIRT1 [96,97]. Indole derivatives may influence chromatin-associated regulation mainly through AhR-dependent co-regulator recruitment, whereas direct effects on HDAC activity require metabolite- and model-specific evidence [91,98]. Redox-sensitive epigenetic enzymes, including TET dioxygenases and JmjC demethylases, can be indirectly modulated through ROS-dependent mechanisms [96]. These observations support the broader view that tryptophan metabolism forms part of a metabolite-driven regulatory network capable of influencing chromatin regulation and stress-responsive transcriptional programs [99].

In parallel, a major fraction of dietary tryptophan in the host is metabolized via the kynurenine pathway, initiated by indoleamine 2,3-dioxygenase 1 (IDO1), indoleamine 2,3-dioxygenase 2 (IDO2), or tryptophan 2,3-dioxygenase (TDO). This pathway generates metabolites such as kynurenine, kynurenic acid, 3-hydroxykynurenine, and quinolinic acid, which play critical roles in immune regulation, neuronal biology, mitochondrial metabolism, and redox biology [100]. Kynurenine itself acts as an endogenous AhR ligand, thereby directly linking host metabolism to AhR-dependent transcriptional and chromatin-associated regulation [93,101]. Through this mechanism, inflammatory signals that induce IDO1 can reshape gene expression programs via AhR activation [101].

A key feature of the kynurenine pathway is its role in de novo NAD^+^ biosynthesis, with quinolinic acid serving as a precursor for NAD^+^ production [97]. NAD^+^ is an essential cofactor for sirtuins, a class of NAD^+^-dependent HDACs that regulate chromatin structure, gene expression, and cellular stress responses [102,103]. Thus, the kynurenine pathway connects metabolic flux to epigenetic regulation through modulation of NAD^+^ availability, activation of sirtuin-mediated histone deacetylation, and regulation of genes involved in oxidative stress and mitochondrial function. Changes in kynurenine pathway activity—such as increased IDO1 expression during inflammation—can therefore lead to context-dependent changes in NAD^+^ availability, sirtuin activity, and chromatin-associated stress responses, affecting redox homeostasis and immune responses [104].

Although derived from distinct sources, indole and kynurenine metabolites converge on a shared regulatory axis involving AhR, Nrf2, and metabolite-sensitive chromatin regulators. Indole derivatives can activate AhR and support barrier-protective or cytoprotective transcriptional programs, favoring transcriptional activation of cytoprotective genes. Kynurenine pathway metabolites integrate inflammatory and metabolic signals, regulating both AhR activity and NAD^+^-dependent epigenetic enzymes such as sirtuins [100]. Both pathways intersect with the Nrf2 antioxidant system, which governs the expression of genes encoding detoxifying and antioxidant enzymes (e.g., HO-1, NQO1). AhR and Nrf2 share coactivators such as CBP/p300 and exhibit coordinated regulation of overlapping gene networks. Recent structural studies have further improved the molecular understanding of ligand-dependent AhR activation and AHR–ARNT–DNA complex formation, providing a stronger framework for interpreting ligand-specific downstream transcriptional effects [105]. Emerging evidence suggests that AhR activation can enhance chromatin accessibility at Nrf2 target genes, thereby facilitating antioxidant responses. In parallel, Nrf2 activity is modulated by epigenetic mechanisms including histone acetylation and methylation, which can be influenced by both indole- and kynurenine-derived signals [106,107]. Taken together, tryptophan-derived metabolites represent a mechanistically diverse signaling network that links microbial and host metabolism to redox regulation through AhR-centered transcriptional control, with chromatin-associated mechanisms likely contributing to the duration and specificity of these responses.

### 2.4. Other Metabolites

Beyond tryptophan-derived metabolites, a growing body of evidence highlights additional classes of microbiota-derived and microbiota-modulated metabolites—including polyphenol-derived compounds, hydrogen sulfide (H_2_S), and lipid mediators—as important, context-dependent regulators of host redox homeostasis. These metabolites act through complementary mechanisms involving modulation of endogenous antioxidant pathways, redox-sensitive signaling, mitochondrial function, inflammatory resolution, and, in selected contexts, chromatin-associated regulation of gene expression [5,99,108].

Dietary polyphenols are extensively metabolized by the gut microbiota into bioactive phenolic acids and smaller aromatic compounds, which often exhibit improved bioavailability and distinct biological activities compared with their parent compounds [109,110]. Key metabolites include urolithins (derived from ellagitannins), phenylpropionic acids, and catechol derivatives.

These microbiota-derived metabolites exert antioxidant effects primarily indirectly, via activation of endogenous defense pathways such as the Nrf2 axis, modulation of inflammatory signaling, and support of mitochondrial function. Direct radical-scavenging activity may contribute in some settings but is unlikely to fully explain their in vivo effects because circulating concentrations are usually low and metabolites differ substantially from their parent polyphenols [111,112]. For example, urolithin A has been shown to improve mitochondrial quality control by inducing mitophagy and supporting mitochondrial function, thereby contributing to cellular resilience [113,114].

Importantly, polyphenol metabolites also function as epigenetic modulators. Several compounds inhibit HDACs and modulate DNA methyltransferase (DNMT) activity, thereby influencing chromatin accessibility and gene expression [115,116]. However, these effects are compound-, dose-, and model-dependent, and the evidence is stronger for some parent polyphenols than for many microbiota-derived metabolites. Therefore, polyphenol-associated epigenetic effects should be presented as plausible and context-dependent rather than uniformly established mechanisms. This distinction is relevant because many studies use purified compounds or parent polyphenols at concentrations that may not reflect the metabolite profiles reached in vivo after microbial conversion.

H_2_S, produced by sulfate-reducing bacteria and microbial cysteine metabolism, is a gaseous signaling molecule with concentration-dependent effects on redox homeostasis [117,118]. Since both host and microbial pathways contribute to H_2_S pools, H_2_S should be described as microbiota-derived and microbiota-modulated rather than exclusively microbiota-derived.

At physiological levels, H_2_S exerts cytoprotective effects by scavenging ROS, enhancing glutathione (GSH) production, and activating Nrf2-dependent antioxidant responses [119,120]. A central mechanism is protein persulfidation, which modifies cysteine residues and regulates protein activity under oxidative stress conditions [121].

H_2_S-mediated persulfidation of Keap1 promotes Nrf2 stabilization and activation, thereby enhancing transcription of antioxidant genes [122]. However, excessive H_2_S production can impair mitochondrial respiration and promote oxidative stress, reflecting its dual role in physiology and pathology [123]. Thus, differences in H_2_S source, local concentration, and exposure time may explain why protective and deleterious effects have both been reported.

Lipid mediators—including eicosanoids, oxidized phospholipids, and specialized pro-resolving mediators (SPMs) such as resolvins and protectins—represent a key interface between redox biology and inflammation [124,125,126]. Most lipid mediators are generated by host enzymatic or non-enzymatic oxidation of polyunsaturated fatty acids, but their production, precursor availability, and signaling are influenced by the gut microbiota. Therefore, they are best described as microbiota-modulated rather than directly microbiota-derived metabolites.

The gut microbiota influences host lipid metabolism and thereby modulates the generation of these mediators [2]. Redox status plays a central role in lipid mediator formation, particularly through the oxidation of polyunsaturated fatty acids. SPMs actively promote the resolution of inflammation while limiting oxidative damage, in part through modulation of mitochondrial ROS production and activation of cytoprotective signaling pathways [2,125]. In addition, oxidized lipid species can regulate transcription factors such as Nrf2 and NF-κB, linking oxidative stress to inflammatory gene expression [127]. Thus, lipid mediators should not be described uniformly as protective; their effects depend on mediator class, biosynthetic pathway, concentration, tissue context, and inflammatory state.

Emerging evidence further suggests that lipid mediators influence epigenetic regulation. For example, certain fatty acid derivatives can alter histone acetylation and chromatin accessibility at inflammatory loci, thereby shaping long-term transcriptional responses [125]. However, direct epigenetic effects of microbiota-modulated lipid mediators remain less well established than their roles in inflammatory signaling and resolution.

Polyphenol-derived metabolites, H_2_S, and lipid mediators represent complementary layers of microbiota–host metabolic communication that converge on redox regulation. While polyphenol metabolites primarily act as epigenetic and transcriptional modulators, H_2_S functions as a redox-sensitive signaling molecule via persulfidation, and lipid mediators serve as downstream effectors linking oxidative stress to inflammation and its resolution.

Collectively, these metabolite classes (1) activate antioxidant pathways (e.g., Nrf2), (2) modulate epigenetic regulators (HDACs, DNMTs, sirtuins), and (3) regulate inflammatory and barrier responses. Disruption of these pathways—through dysbiosis, dietary imbalance, or chronic inflammation—can impair redox homeostasis and contribute to the development of metabolic, inflammatory, and neoplastic diseases [99].

Microbiota-derived and microbiota-modulated metabolites beyond tryptophan—including polyphenol derivatives, H_2_S, and lipid mediators—play essential roles in the regulation of redox homeostasis. By integrating direct antioxidant effects with signaling and epigenetic mechanisms, these metabolites form a complex regulatory network that maintains cellular and tissue integrity. Since much of the available mechanistic evidence is derived from gastrointestinal or intestinal epithelial and immune cell models, systemic effects should be interpreted cautiously. Extraintestinal effects are discussed only where supported by specific experimental or clinical evidence, and their generalizability across tissues remains context-dependent. However, the level of evidence and translational maturity differ substantially between metabolite classes. To support comparison across the major microbiota-derived and microbiota-modulated metabolite groups, Table 1 summarizes the predominant evidence base, main mechanistic support, and current translational interpretation for each class (Table 1).

## 3. Epigenetic Regulation of Redox Homeostasis

**Figure 2:**
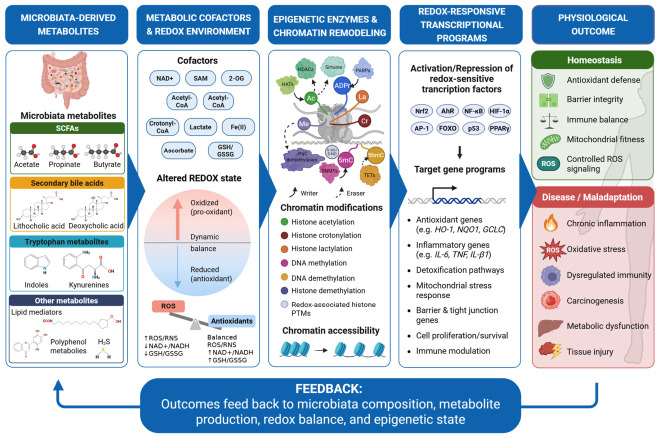
Integrated framework linking microbiota-derived metabolites, redox biology, epigenetic regulation, and physiological outcomes. Microbiota-derived and microbiota-modulated metabolites, including short-chain fatty acids (SCFAs), secondary bile acids, tryptophan metabolites, polyphenol-derived metabolites, H_2_S), and lipid mediators, influence host physiology through multiple interconnected mechanisms. These metabolites shape the intracellular metabolic and redox environment by affecting cofactors such as NAD^+^, SAM, 2-OG, acetyl-CoA, crotonyl-CoA, lactate, Fe(II), ascorbate, and the glutathione redox couple (GSH/GSSG). In turn, these factors regulate epigenetic enzymes and chromatin-remodeling processes, including histone acetylation, crotonylation, lactylation, DNA methylation and demethylation, histone demethylation, redox-associated histone PTMs, and chromatin accessibility. These chromatin-associated changes modulate redox-responsive transcriptional programs governed by transcription factors such as Nrf2, AhR, NF-κB, HIF-1α, AP-1, FOXO, p53, and PPARγ. Depending on metabolite composition, concentration, cell type, tissue context, and disease state, these mechanisms can promote homeostasis, including antioxidant defense, barrier integrity, immune balance, mitochondrial fitness, and controlled ROS signaling, or contribute to maladaptive outcomes such as chronic inflammation, oxidative stress, dysregulated immunity, carcinogenesis, metabolic dysfunction, and tissue injury. These outcomes further feed back on microbiota composition, metabolite production, redox balance, and the epigenetic state, forming a dynamic and bidirectional microbiota–metabolite–redox–epigenetic axis. Abbreviations: 2-OG, 2-oxoglutarate; AhR, aryl hydrocarbon receptor; AP-1, activator protein 1; CoA, coenzyme A; Fe(II), ferrous iron; FOXO, forkhead box O; GSH, reduced glutathione; GSSG, oxidized glutathione; HIF-1α, hypoxia-inducible factor 1-alpha; NAD^+^, nicotinamide adenine dinucleotide; NF-κB, nuclear factor kappa-light-chain-enhancer of activated B cells; Nrf2, nuclear factor erythroid 2-related factor 2; PPARγ, peroxisome proliferator-activated receptor gamma; PTMs, post-translational modifications; ROS, reactive oxygen species; SAM, S-adenosylmethionine. Created in Biorender https://www.biorender.com/ (accessed on 2 June 2026).

Redox homeostasis and epigenetic regulation are intimately connected. Cellular redox conditions influence the activity of chromatin-modifying enzymes, while epigenetic mechanisms determine how efficiently cells express antioxidant, inflammatory, metabolic, and stress-response genes. This creates a bidirectional relationship in which redox changes can reshape chromatin regulation, and chromatin regulation can, in turn, affect the cellular response to oxidative stress and electrophilic injury [128]. This interplay is relevant at several levels. Redox-dependent changes in metabolite availability can alter the activity of enzymes that write, erase, or interpret epigenetic modifications. In addition, oxidative conditions can modify catalytic cofactors and, in some cases, directly affect chromatin regulators themselves through redox-sensitive post-translational modifications. Therefore, redox imbalance is not only an endpoint of cellular dysfunction, but also a potential driver of chromatin remodeling and persistent transcriptional reprogramming (Figure 2) [128,129]. In the context of microbiota–host interactions, this concept is particularly relevant because microbiota-derived and microbiota-modulated metabolites can influence both the intracellular redox environment and the availability of epigenetic cofactors, thereby linking microbial metabolism to redox-sensitive gene regulation. However, in many studies, it remains difficult to separate direct effects on epigenetic enzymes from indirect consequences of altered metabolism, inflammatory signaling, or oxidative stress. This limitation should be considered when interpreting metabolite-associated changes in chromatin state or gene expression.

### 3.1. Histone Deacetylases (HDACs), Histone Acetyltransferases (HATs), and Chromatin Dynamics in Redox Control

One of the clearest links between redox signaling and epigenetic regulation is detected at the level of histone acetylation. The balance between HATs and HDACs is dynamic and responsive to inflammatory and oxidative cues, allowing chromatin accessibility to shift in parallel with stress signaling. In this way, histone acetylation provides a relatively rapid interface through which redox changes can be translated into altered gene expression (Table 2) [129].

Oxidative stress has been shown to disturb this balance. In inflammatory contexts, altered HAT and HDAC activity can reshape transcriptional programs, including NF-κB-dependent inflammatory genes, but the outcome is context-dependent and may either amplify or restrain inflammation depending on cell type, stimulus, and chromatin state. This context dependency is important because increased histone acetylation is often interpreted as transcriptionally activating, but its functional consequences depend on the genomic locus, the transcription factors recruited, and the inflammatory environment. Thus, changes in HAT or HDAC activity cannot be interpreted in isolation from the broader signaling context. At the same time, some HDACs are direct targets of redox-derived electrophiles. Class I HDACs, particularly HDAC1, HDAC2, and HDAC3, are sensitive in this regard. Reactive lipid peroxidation products such as 4-hydroxy-2-nonenal and 15-deoxy-Δ12,14-prostaglandin J2 can modify conserved cysteine residues in HDAC1, HDAC2, and HDAC3, leading to loss of activity and derepression of cytoprotective genes such as HO-1 and heat shock protein 70 (HSP70) [129].

A different situation applies to class III HDACs, the sirtuins, which are NAD^+^-dependent deacetylases and deacylases rather than zinc-dependent HDACs. Their activity depends on NAD^+^ availability and the NAD^+^/NADH ratio, linking them directly to mitochondrial function, energy metabolism, and cellular redox state. Thus, changes in oxidative metabolism, inflammation, or nutrient availability can influence sirtuin-dependent chromatin regulation and stress-response programs. A related principle applies to poly(ADP-ribose) polymerases (PARPs), which also consume NAD^+^ and participate in DNA damage responses, chromatin remodeling, and stress-associated transcriptional regulation. Together, these relationships show that redox control of histone acetylation is mediated not only through signaling pathways, but also through core metabolic cofactors and redox-sensitive enzyme regulation [128,129].

### 3.2. DNA Methylation and Redox-Sensitive Methylation Capacity

DNA methylation represents a second major interface between redox biology and epigenetic control. Although often discussed as a relatively stable epigenetic modification, DNA methylation remains closely connected to metabolism through its dependence on S-adenosylmethionine (SAM), the universal methyl donor for DNA and histone methyltransferases. SAM synthesis is influenced by one-carbon metabolism, methionine cycle activity, and glutathione-dependent redox balance, which creates a direct biochemical connection between oxidative conditions and cellular methylation capacity [128,138]. Since glutathione synthesis and SAM generation are metabolically linked through sulfur amino acid metabolism, oxidative stress can shift methyl donor availability and thereby influence DNA and histone methylation potential.

This connection is functionally relevant, since oxidative stress has been associated with broader changes in DNA methylation patterns as well as with concurrent alterations in histone modifications. Such changes may include both global alterations in methylation capacity and locus-specific DNA methylation changes at genes involved in inflammation, antioxidant defense, mitochondrial function, and stress adaptation. These observations support the view that redox imbalance can actively reshape the epigenetic state of the cell rather than simply arise downstream of transcriptional dysregulation [129]. However, the direction and functional consequence of redox-associated DNA methylation changes are highly context-dependent and may vary according to cell type, duration of oxidative stress, inflammatory state, and availability of methyl donors and reducing equivalents. Moreover, most studies report associations between oxidative stress and altered methylation patterns, whereas direct causal links between specific microbiota-derived metabolites, DNA methylation changes, and redox outcomes remain less well established.

### 3.3. Redox-Sensitive Demethylation: TET and JmjC Enzymes

A particularly important mechanistic bridge between redox metabolism and epigenetic regulation is formed by the Fe(II)- and 2-oxoglutarate-dependent dioxygenases, including the TET family of DNA cytosine dioxygenases and the JmjC-domain histone demethylases. These enzymes require molecular oxygen, reduced iron, and 2-oxoglutarate (2-OG) for catalysis, making them highly sensitive to oxygen availability, iron redox state, and metabolic cofactor levels [129,135,136,139]. The TET enzymes mediate the stepwise oxidation of 5-methylcytosine to 5-hydroxymethylcytosine, 5-formylcytosine, and 5-carboxylcytosine, thereby contributing to active and passive DNA demethylation pathways. Because these reactions depend on catalytic Fe(II), oxidizing conditions may impair TET activity, at least in part, by reducing the availability of catalytically active Fe(II) and altering the redox environment required for dioxygenase activity (Table 2). This provides a plausible mechanism by which oxidative stress may alter DNA methylation landscapes and downstream transcriptional programs involved in differentiation, adaptation, and antioxidant defense [129,135,136,139].

TET activity is also shaped by metabolite availability. 2-OG supports catalysis, whereas succinate and fumarate can restrain Fe(II)/2-OG-dependent dioxygenase activity by antagonizing 2-OG-dependent enzymatic reactions. Conversely, reducing cofactors such as ascorbate can support TET function by helping maintain iron in the reduced Fe(II) state and by promoting TET-dependent cytosine oxidation (Table 2) [129,135,136,139].

A similar biochemical mechanism applies to the JmjC-domain histone demethylases, which remove methyl groups from histone lysine residues through related Fe(II)/2-OG-dependent mechanisms (Table 2). Since these enzymes are sensitive to oxygen tension and redox conditions, they have been discussed as potential chromatin-based oxygen sensors. This is particularly relevant in situations where hypoxia and oxidative stress coexist, such as in inflamed or metabolically compromised tissues [136,140]. Hypoxia can limit JmjC activity by reducing oxygen availability, whereas oxidative stress may interfere with demethylase activity by altering Fe(II) availability, ascorbate-dependent redox buffering, or the broader intracellular redox state. These mechanisms may contribute to the accumulation of specific histone methylation states under pro-oxidant or hypoxic conditions [129,132,136,141].

At the same time, certain JmjC demethylases themselves participate in the regulation of redox homeostasis. Lysine demethylase 2B (KDM2B), also known as Ndy1, has been reported to promote cellular resistance to oxidative stress and to support the expression of antioxidant and metabolism-associated genes, including NQO1, peroxiredoxin 4 (PRDX4), and aminoadipate-semialdehyde synthase (AASS), thereby contributing to cellular protection against oxidative stress (Table 3) [125,129,137].

### 3.4. Histone Modifications Beyond Acetylation: Acylations and Redox-Associated Post-Translational Modifications (PTMs)

Although histone acetylation is the best-established example of metabolically linked chromatin regulation, the spectrum of relevant histone modifications is broader. Histone acylations, including crotonylation and lactylation, connect intermediary metabolism to chromatin state because they depend on intracellular acyl-CoA or metabolite-derived donor pools. However, for several of these newer modifications, their functional role in redox regulation is still emerging, and in many cases it remains difficult to distinguish causative effects from broader changes in cellular metabolism. Histone crotonylation, in particular, has been associated with active promoters and enhancers and linked to genes involved in metabolism and stress responses [45,138].

Histone crotonylation is supported by crotonyl-CoA availability and can be catalyzed by p300/CBP-containing acetyltransferase complexes, which also possess crotonyltransferase activity. This provides a direct biochemical route through which changes in acyl-CoA metabolism can influence transcriptional activity [45]. A related principle applies to acetyl-CoA, which supplies the substrate for HAT-mediated histone acetylation and thereby links glycolysis, fatty acid metabolism, and the tricarboxylic acid cycle to chromatin accessibility. Lactate has likewise emerged as an epigenetically relevant metabolite, especially in inflammatory and immune contexts, where lactate-derived histone lactylation can contribute to transcriptional regulation during hypoxia, bacterial challenge, macrophage activation, and cellular stress adaptation [138,144]. However, histone lactylation remains an emerging field, and its functional consequences are likely to be cell type-, locus-, and disease-context dependent.

Oxidative stress can also generate less conventional histone post-translational modifications, including carbonylation, glutathionylation, and nitrosylation. These redox-associated histone modifications broaden the mechanisms through which oxidative stress can influence chromatin architecture, because they may alter histone-DNA interactions or protein recruitment independently of classical acetylation and methylation pathways [129,138]. Unlike enzymatically regulated histone acylations, several redox-associated PTMs may arise through direct chemical modification by reactive oxygen, nitrogen, or electrophilic species. Their effects on gene expression are therefore often context-dependent and may reflect both regulated redox signaling and oxidative damage.

Together, these findings indicate that chromatin can integrate metabolic and redox information through multiple layers of histone modification. While acetylation and crotonylation provide relatively well-characterized links between acyl-CoA metabolism and transcriptional activation, lactylation and redox-associated histone PTMs represent emerging mechanisms that may contribute to stress-responsive and inflammation-associated gene regulation.

### 3.5. Metabolites as Epigenetic Cofactors

A central conclusion emerging from this field is that many epigenetic enzymes depend directly on metabolite availability. NAD^+^ regulates sirtuins and PARPs, SAM supports methylation reactions, and 2-OG is required for TET and JmjC enzymes. In parallel, redox-sensitive systems involving glutathione, catalytic iron, and reducing cofactors such as ascorbate influence the biochemical environment in which these enzymes operate. This makes the epigenetic machinery highly responsive to metabolic flux and redox conditions (Table 4) [129]. If chromatin regulation depends strongly on metabolite availability and redox-sensitive cofactors, then microbiota-derived metabolites should not be considered only as extracellular signaling molecules. They may also influence the intracellular biochemical conditions that determine chromatin state, enzymatic activity, and transcriptional responsiveness. In this sense, the epigenetic machinery can be regarded as a metabolic integration platform through which redox-relevant microbial signals acquire more sustained biological effects [138].

### 3.6. Functional Implications for Stress Adaptation and Disease

The close coupling between redox control and epigenetic regulation has important implications for both physiology and disease. Under physiological conditions, it allows cells to adjust antioxidant defenses, inflammatory programs, metabolic pathways, and repair mechanisms in a flexible and context-dependent manner. Such redox-sensitive chromatin regulation can support cellular adaptation by enabling transient transcriptional responses to oxidative, electrophilic, metabolic, or inflammatory stress. Under chronic stress conditions, however, the same mechanisms may contribute to more persistent and maladaptive transcriptional states that stabilize inflammation, impair tissue function, or promote degenerative and neoplastic processes [136,145,146].

This distinction between adaptive and maladaptive chromatin remodeling is particularly relevant in tissues exposed to fluctuating microbial, dietary, and inflammatory cues, such as the intestinal mucosa [5,145,146]. In this environment, metabolites derived from or modulated by the microbiota can influence redox balance, mitochondrial metabolism, inflammatory signaling, and the availability of epigenetic cofactors [7,145,146]. As a result, microbial metabolites may help maintain stress resilience and barrier integrity under homeostatic conditions [7,147], but may also contribute to epigenetic dysregulation when dysbiosis, chronic inflammation, or sustained oxidative stress persist [5,148].

Taken together, these findings identify epigenetic regulation as a central mechanistic interface between metabolism and redox biology [145,146,149]. This framework is particularly relevant to understanding why microbiota-derived and microbiota-modulated metabolites may influence host redox homeostasis not only through receptor-mediated signaling, but also by shaping chromatin organization, enzyme activity, and the transcriptional potential of stress-responsive genes [5,145,146]. Thus, the microbiota–metabolite–redox–epigenetic axis provides a useful conceptual framework for understanding how environmental and metabolic signals are converted into either protective adaptation or disease-promoting transcriptional programs [5,145,148].

## 4. Linking Microbial Metabolism, Epigenetics, and Redox Signaling

Microbiota-derived metabolites influence host cells through overlapping receptor-mediated, metabolic, redox-sensitive, and chromatin-associated mechanisms [128,129,138,145,146]. Since these processes interact rather than operate independently, their biological effects are best understood within an integrated regulatory framework. In this framework, microbial metabolites act as upstream inputs that shape signaling pathways, redox state, cofactor availability, and chromatin-associated regulation, thereby influencing stress-responsive transcriptional programs and biological outcomes in a context-dependent manner.

Together, these considerations support an integrated view of microbial metabolite signaling, in which metabolic cues, epigenetic regulation, and redox-responsive pathways are closely linked. This framework is particularly useful for interpreting the metabolite–epigenetic–redox axis, crosstalk between Nrf2, NF-κB and AhR, and the consequences of these interactions in different cells and contexts [99].

### 4.1. The Metabolite–Epigenetic–Redox Axis

The metabolite–epigenetic–redox axis describes how chemically diverse microbial metabolites converge on host stress-response programs through distinct but interconnected mechanisms. At the initial level, metabolites act through membrane or nuclear receptors, intracellular metabolism, or the availability of epigenetically relevant cofactors. These inputs shape chromatin accessibility and transcriptional responsiveness and thereby influence antioxidant defense, inflammatory activation, epithelial barrier integrity, and immune cell function [99,138,150,151].

Epigenetic regulation is the central intermediate layer of this axis. As described in Chapter 3, the epigenetic machinery is sensitive to the cellular redox status and the metabolic state. DNA methyltransferases, histone acetyltransferases, deacetylases, and demethylases all depend on specific metabolic substrates, cofactors, or catalytic conditions, meaning they are responsive to metabolite availability and redox imbalance. Therefore, histone acetylation, DNA methylation, TET- and JmjC-dependent demethylation, and emerging histone acylations provide multiple routes through which microbial metabolites may influence the accessibility, amplitude, and persistence of stress-responsive gene programs [132,138,151,152]. Redox signaling constitutes the functional output layer of this system, but it also feeds back into the system. Cellular redox homeostasis is shaped by the coordinated regulation of antioxidant defenses, mitochondrial function, inflammatory mediators, and detoxification pathways [9,10,153]. Meanwhile, oxidative stress can modulate the activity of epigenetic enzymes through changes in cofactor availability and oxidative post-translational modifications of chromatin-associated proteins and histones. Experimental studies have demonstrated that oxidative stress alters global histone modification patterns and DNA methylation. Specific redox-dependent chromatin changes include the S-glutathionylation of histone H3 and histone carbonylation [132,154]. Conversely, epigenetic regulatory mechanisms can alter the expression of antioxidant and stress-response genes. Redox-derived electrophiles, for example, can inactivate class I HDACs and derepress cytoprotective genes such as HO-1 and HSP70, whereas redox-sensitive histone demethylase pathways can promote antioxidant gene expression and cellular protection against oxidative stress. This reciprocal organization is a defining feature of redox-sensitive epigenetic regulation [132].

Importantly, the metabolite–epigenetic–redox axis is not a linear cascade, but rather a dynamic, bidirectional system. Microbial metabolites shape the metabolic and signaling context in which chromatin regulation occurs, while redox perturbations feed back into the epigenetic machinery and thereby alter how cells transcriptionally respond to microbiota-derived metabolic signals. This helps to explain why the same metabolite class may have protective or detrimental effects, depending on concentration, cellular state, tissue environment, and disease context. Taken together, the metabolite–epigenetic–redox axis provides a conceptual bridge between the descriptive catalogue of microbial metabolites and the mechanistic complexity of host stress responses [138,150,151].

### 4.2. Crosstalk Between the Nuclear Factor Erythroid 2-Related Factor 2 (Nrf2), the Nuclear Factor Kappa-Light-Chain-Enhancer of Activated B Cells (NF-κB), and the Aryl Hydrocarbon Receptor (AhR)

**Figure 3:**
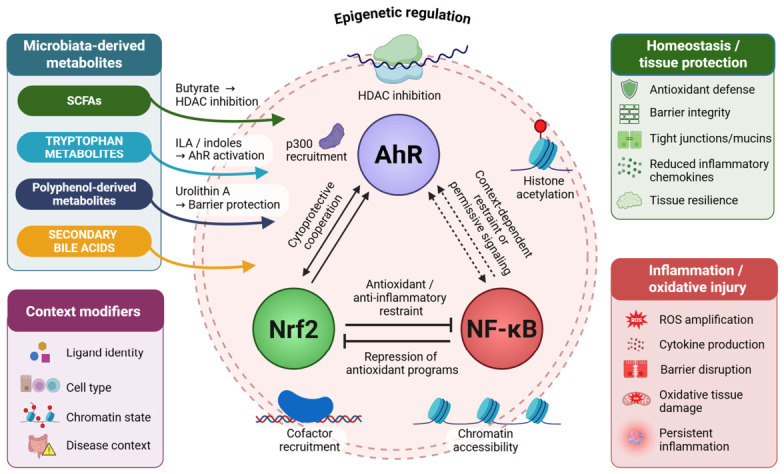
Context-dependent integration of microbiota-derived metabolite signaling by AhR, Nrf2, and NF-κB through epigenetic regulation. Microbiota-derived metabolites, including short-chain fatty acids (SCFAs), tryptophan metabolites, polyphenol-derived metabolites, and secondary bile acids, influence host responses through distinct but partially overlapping mechanisms. These signals converge on the transcriptional hubs AhR, Nrf2, and NF-κB, whose interplay is shaped by chromatin accessibility, histone acetylation, HDAC inhibition, and cofactor recruitment. The functional outcome depends on contextual modifiers such as ligand identity, cell type, chromatin state, and disease setting. Under permissive conditions, this network promotes antioxidant defense, barrier integrity, reduced inflammatory signaling, and tissue resilience, whereas under context-dependent or dysregulated conditions it may contribute to cytokine production, barrier disruption, oxidative tissue injury, and persistent inflammation. Abbreviations: AhR, aryl hydrocarbon receptor; HDAC, histone deacetylase; NF-κB, nuclear factor kappa-light-chain-enhancer of activated B cells; Nrf2, nuclear factor erythroid 2-related factor 2; SCFAs, short-chain fatty acids. Created in Biorender https://BioRender.com (accessed on 29 May 2026).

A central integrative layer of this network is the crosstalk between Nrf2, NF-κB, and AhR, which links antioxidant defense, inflammatory signaling, and microbial metabolite sensing (Figure 3).

Among these pathways, Nrf2 is a central regulator of antioxidant defense, and several microbiota-derived metabolites have been shown to enhance redox-protective gene programs through Nrf2-dependent mechanisms [155].

NF-κB is a major transcriptional regulator of inflammatory gene expression. It controls the induction of cytokines, chemokines, and other mediators required for host defense, but can also amplify tissue injury when activation becomes excessive or persistent [153]. In many contexts, Nrf2 and NF-κB are functionally antagonistic, with Nrf2-driven antioxidant responses limiting ROS-dependent inflammatory amplification, whereas NF-κB activation promotes a pro-oxidant milieu that reinforces inflammatory injury [156,157]. This relationship extends beyond indirect redox effects. Experimental work has shown that NF-κB p65 can cooperate with HDAC3 to repress Nrf2-ARE activity, thereby suppressing antioxidant gene expression and promoting oxidative stress-associated injury [158]. Conversely, Nrf2 activation can restrain inflammatory signaling and oppose NF-κB-driven transcriptional programs, making the balance between these pathways a key determinant of whether oxidative stress is resolved through adaptive cytoprotection or progresses toward persistent inflammation and tissue damage [157].

AhR has a particularly important role within this network because it functions as a metabolite-responsive transcription factor. Many microbiota-derived tryptophan metabolites act as endogenous AhR ligands, allowing microbial metabolism to directly influence host transcriptional programs. However, AhR activity extends well beyond canonical xenobiotic signaling. It intersects with both antioxidant and inflammatory pathways and has been implicated in the regulation of barrier integrity, immune differentiation, and oxidative stress responses. A growing number of studies support the view that AhR cooperates with Nrf2 in the induction of cytoprotective programs, while its relationship with NF-κB is more context-dependent and can be either restraining or permissive depending on ligand identity, cell type, and inflammatory state [159]. This variability should be considered when comparing studies, because different AhR ligands or inflammatory models may produce distinct, and sometimes not directly comparable, transcriptional outcomes.

Beyond functional overlap, recent work suggests that AhR–Nrf2 cooperation may involve a more structured, chromatin-associated transcriptional mechanism. The recently described AHR–NRF2–JDP2 gene battery proposes that the transcriptional regulator JDP2 facilitates the movement of AhR–Nrf2 complexes between xenobiotic and antioxidant response elements in a spatiotemporal manner during ligand-induced transcriptional activation. This model provides a plausible, chromatin-based mechanism through which AhR–Nrf2 cooperation could contribute to more sustained cytoprotective gene expression [95].

The interaction between AhR and NF-κB is particularly context-dependent. In intestinal epithelial cells, microbial tryptophan metabolites can activate AhR and thereby attenuate inflammatory transcriptional programs linked to NF-κB, glycolysis, and hypoxia-associated signaling. It has been shown that ILA reduces inflammatory chemokine production and alters epithelial–macrophage crosstalk, thereby limiting recruitment of inflammatory macrophages and supporting intestinal homeostasis [158,160]. Further related work indicates that ILA can improve barrier integrity via the AhR/Nrf2/NF-κB axis by suppressing NF-κB-dependent inflammatory responses and promoting Nrf2-activated barrier-protective programs [161]. These findings are important because they show that AhR does not simply act as an isolated metabolite sensor, but can integrate microbial metabolite signals in a way that simultaneously restrains inflammation, promotes antioxidant defense, and strengthens epithelial barrier function.

Microbiota-derived metabolites can also shift the balance among Nrf2, NF-κB, and AhR through epigenetic and chromatin-associated mechanisms. Butyrate, a SCFA produced by commensal bacteria, provides a particularly clear example. Experimental studies have shown that butyrate suppresses NF-κB-dependent inflammatory responses through histone deacetylase inhibition, while at the same time reshaping chromatin accessibility and barrier-associated transcriptional programs. In intestinal epithelial cells, butyrate has been shown to inhibit the HDAC8/NF-κB pathway, enhance the Chloride anion exchanger/Down-regulated in adenoma (DRA) expression, and improve epithelial barrier integrity [162]. In monocytes and macrophages, butyrate-dependent suppression of mucosal inflammation has been linked to HDAC3 inhibition, increased histone acetylation, and reduced expression of pro-inflammatory mediators such as interleukin (IL)-6 and tumor necrosis factor (TNF) [163,164]. These studies provide a mechanistic basis for how microbiota-derived metabolites can restrain inflammatory transcriptional programs while simultaneously promoting barrier protection and tissue homeostasis.

The transcriptional output of this network is likely to be largely shaped by the chromatin state of target genes. The biological consequences of Nrf2, NF-κB, and AhR activation depend on more than ligand binding or oxidative stress intensity; promoter accessibility, histone acetylation, methylation status, and cofactor recruitment also play a role. Experimental work has demonstrated that AhR and Nrf2 can cooperate to recruit p300 to target promoters, thereby restoring histone H3 acetylation and modulating antioxidant gene expression in a chromatin-dependent manner [165]. In this framework, microbial metabolites influence not only signaling pathways themselves, but also the chromatin environment in which these pathways act. This means that the Nrf2–NF-κB–AhR triad is best understood as part of a shared regulatory network whose output is determined by both signaling activity and chromatin state [165].

Taken together, Nrf2, NF-κB, and AhR form a central signaling triad through which microbiota-derived metabolites influence redox homeostasis, inflammatory tone, and cellular adaptation. The balance between these pathways appears to be a major determinant of whether microbial metabolite signaling promotes tissue protection and homeostasis or contributes to persistent inflammation and oxidative injury. Importantly, this balance is not fixed, but is dynamically shaped by metabolite composition, chromatin state, cell type, and disease context [159].

### 4.3. Cell Type-Specific Integration of Microbial Metabolite Signaling

Microbiota-derived metabolites are not interpreted uniformly across host tissues. Although many of these compounds converge on shared regulatory nodes such as Nrf2, NF-κB, AhR, and redox-sensitive epigenetic enzymes, their downstream effects depend strongly on cellular identity. Differences in receptor expression, metabolic requirements, chromatin accessibility, and baseline transcriptional programs determine how individual cell types respond to microbial metabolite exposure. This concept is supported by work showing that diet–microbiota interactions can reshape host epigenetic programming across multiple tissues, rather than in a single cellular compartment alone [145].

In intestinal epithelial cells, microbiota-derived metabolites primarily influence barrier maintenance, redox defense, and local stress adaptation. Tryptophan-derived microbial metabolites can improve intestinal barrier integrity through AhR-dependent mechanisms [86]. More specifically, microbial ILA has been linked to epithelial barrier protection through the AhR/Nrf2/NF-κB axis [161], while urolithin A, a gut microbial metabolite derived from ellagitannins, has been reported to enhance mucin-associated barrier defense through AhR- and Nrf2-related mechanisms [166]. Together, these findings support the concept that epithelial responses are shaped by coordinated metabolite–redox signaling rather than by isolated pathways.

In myeloid cells, the consequences are more directly linked to inflammatory tone and immune activation. A landmark study showed that the microbial metabolite butyrate regulates intestinal macrophage function through histone deacetylase inhibition, rendering these cells less responsive to inflammatory stimulation and thereby providing a clear example of cell type-specific epigenetic immunoregulation [164]. More recent work has extended this concept by showing that butyrate can suppress mucosal inflammation in monocytes and macrophages predominantly through HDAC3 inhibition, accompanied by increased histone acetylation and reduced pro-inflammatory mediator expression [163]. In epithelial–immune crosstalk models, microbial ILA has also been shown to reduce inflammatory chemokine production and limit recruitment of inflammatory macrophages, illustrating that microbial metabolites may shape immune cell behavior both directly and indirectly through neighboring epithelial cells [160].

In T cells, microbiota-derived metabolites strongly influence differentiation and functional polarization. Two seminal Nature papers demonstrated that commensal bacteria-derived metabolites, particularly butyrate and other SCFAs, promote the differentiation of regulatory T cells, thereby linking microbial metabolism directly to adaptive immune regulation [40,43]. These studies are particularly relevant in the present context since they show that microbial metabolites do not merely dampen inflammation in a general sense but can alter lineage-defining immune programs in a cell type-specific manner. Such effects are highly compatible with the idea that metabolic and epigenetic regulation together determine whether immune cells adopt inflammatory or regulatory phenotypes.

A further layer of complexity arises from the fact that different cell types are not only exposed to different metabolite concentrations, but also differ in their metabolic wiring, cofactor availability, and chromatin state. This means that the same metabolite may support antioxidant defense and tissue protection in one compartment while exerting weaker, altered, or even adverse effects in another [99,167,168]. Epithelial cells, for example, may primarily translate microbial metabolites into barrier-protective and antioxidant programs, whereas immune cells may respond by altering cytokine production, inflammatory activation, or differentiation trajectories. These cell type-specific responses are consistent with the broader view that gut microbial metabolites act as multi-kingdom intermediates whose biological meaning depends on the host context in which they are interpreted [99].

### 4.4. Context Dependency in Homeostasis and Disease

**Figure 4:**
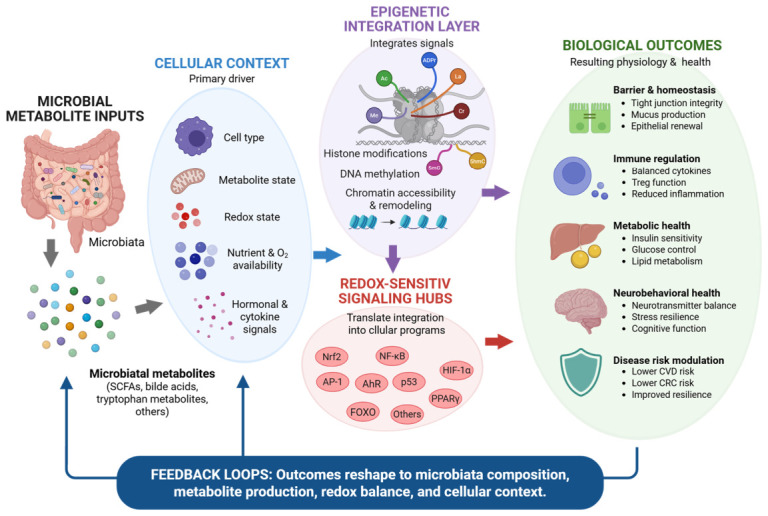
Conceptual model of microbiota–metabolite–epigenetic integration in redox-sensitive physiology and disease modulation. Microbial metabolite inputs are interpreted within a cell-specific context defined by cell type, metabolic state, redox status, nutrient and oxygen availability, and hormonal or cytokine signals. These contextual cues converge on an epigenetic integration layer that includes histone modifications, DNA methylation, chromatin accessibility, and chromatin remodeling. In parallel, redox-sensitive signaling hubs, including Nrf2, NF-κB, AhR, HIF-1α, AP-1, FOXO, p53, PPARγ, and additional transcriptional regulators, translate these integrated signals into cellular gene programs. The resulting biological outcomes include regulation of epithelial barrier integrity, mucus production, epithelial renewal, immune balance, metabolic health, neurobehavioral resilience, and disease risk modulation. Feedback loops from these outcomes can further reshape microbiota composition, metabolite production, redox balance, and cellular context, establishing a dynamic and bidirectional microbiota–metabolite–epigenetic–redox axis. Abbreviations: AhR, aryl hydrocarbon receptor; AP-1, activator protein 1; FOXO, forkhead box O; HIF-1α, hypoxia-inducible factor 1-alpha; NF-κB, nuclear factor kappa-light-chain-enhancer of activated B cells; Nrf2, nuclear factor erythroid 2-related factor 2; PPARγ, peroxisome proliferator-activated receptor gamma; ROS, reactive oxygen species. Created in Biorender. https://BioRender.com (accessed on 2 June 2026).

The biological effects of microbiota-derived and microbiota-modulated metabolites depend strongly on concentration, exposure duration, tissue environment, cell type, inflammatory state, and disease context. Thus, the same metabolite class may support epithelial barrier integrity, antioxidant defense, immune tolerance, or metabolic adaptation under homeostatic conditions, but may contribute to maladaptive signaling, oxidative stress, or inflammatory persistence when dysbiosis, chronic inflammation, or metabolic disruption alters the metabolite landscape and host-response state (Figure 4) [86,99,145,164].

## 5. Clinical and Translational Implications

The mechanistic links between microbiota-derived metabolites, epigenetic regulation, and redox signaling may have important clinical implications, although their translational relevance still needs to be defined more precisely. Altered microbial community structure is frequently accompanied by changes in the production, distribution, and host exposure of bioactive metabolites, including SCFAs, secondary bile acids, and tryptophan-derived compounds. These metabolite shifts are increasingly recognized not merely as secondary consequences of disease, but as functionally relevant components of disease-associated host–microbiota dysregulation. However, the strength of evidence differs across settings: experimental studies provide mechanistic support for several pathways, whereas many human studies remain associative and do not yet establish causality. Clinical translation, therefore, requires moving beyond descriptive taxonomic dysbiosis and toward a framework that also considers metabolite availability, host response capacity, and redox-epigenetic context. This broader view is supported by microbiome-to-host translational perspectives and by studies showing that diet–microbiota interactions can influence host epigenetic programming across tissues [169,170].

### 5.1. Dysbiosis and Altered Metabolite Profiles

Dysbiosis is best understood not only as a shift in microbial composition, but also as a disturbance in the biochemical interface between the microbiota and the host. In many disease settings, changes in microbial diversity and taxonomic structure are accompanied by altered SCFA production, disturbed bile acid transformation, and dysregulated tryptophan metabolism. This matters because these metabolite classes do not simply reflect microbial activity; they directly influence epithelial integrity, inflammatory signaling, redox balance, and chromatin-associated gene regulation. Clinical phenotypes associated with dysbiosis may therefore arise in part from a reorganization of the metabolite landscape rather than from compositional changes alone [14,171].

One of the clearest examples comes from inflammatory bowel disease (IBD). Multiple studies indicate that IBD is associated with depletion of SCFA-producing taxa and with quantitative reductions in fecal SCFAs, particularly during active disease. In parallel, ulcerative colitis and Crohn’s disease have been linked to altered bile acid pools, including reduced levels of anti-inflammatory secondary bile acids and impaired microbial bile acid conversion. This pattern suggests that dysbiosis is accompanied by the loss of metabolites that normally contribute to epithelial barrier maintenance, inflammatory restraint, and redox adaptation [172,173,174].

Altered tryptophan metabolism provides another clinically important example. A multi-omics study from 2024 showed that gut microbiota dysbiosis was associated with altered tryptophan metabolism together with dysregulated inflammatory responses in severe COVID-19, illustrating how disease-associated microbiome disruption can be linked to a coordinated shift in metabolite and immune profiles. Although the disease context differs from chronic intestinal disorders, the study is highly relevant conceptually because it shows that dysbiosis can be accompanied by measurable perturbations in metabolite pathways with plausible consequences for host inflammatory and redox responses [171].

Comparable principles are now emerging in metabolic disease. In 2025, a Nature Metabolism study identified serum microbial aromatic amino acid metabolites associated with body fat accumulation in a large longitudinal cohort and further showed protective effects of selected metabolites in experimental obesity models. These findings support the idea that dysbiosis-associated alterations in microbial metabolite output can track with systemic metabolic phenotypes and may help distinguish protective from maladaptive metabolite signatures [169].

The same logic also applies to colorectal tumorigenesis, where dysbiosis is frequently associated with shifts away from protective metabolites and toward metabolites with pro-inflammatory, pro-oxidant, or tumor-promoting potential, including specific secondary bile acids and sulfur-containing compounds. While the precise direction and magnitude of these changes depend on disease stage, diet, and microbial ecology, the overall pattern supports the view that metabolite dysregulation is one of the clinically relevant outputs of dysbiosis rather than a peripheral feature [175,176].

From a translational perspective, these observations suggest that disease-associated dysbiosis should be assessed at both the compositional and metabolite levels. Taxonomic profiling alone may not adequately capture the functional consequences of microbiome disruption, whereas combined microbiome–metabolome approaches are more likely to identify clinically meaningful signatures. This is particularly important in disorders where the same nominal metabolite class may be protective in one context but insufficient, redistributed, or maladaptive in another. A clinically useful model of dysbiosis, therefore, has to incorporate not only which microbes are altered, but also which metabolites are lost, enriched, or functionally reinterpreted by the host [170,171,177].

### 5.2. Metabolite and Epigenetic Signatures as Candidate Biomarkers

The altered metabolite profiles associated with dysbiosis may serve not only as indicators of disturbed host–microbiota interactions, but also as clinically informative biomarker candidates. When combined with host epigenetic readouts, such signatures may capture both the functional output of the microbiota and the regulatory response of the host. Microbiota-derived metabolites are attractive biomarker candidates because they reflect microbial activity, substrate utilization, host exposure, and pathway engagement more directly than taxonomic profiling alone, whereas epigenetic readouts may indicate whether these signals have been translated into more stable host transcriptional programs (Table 5) [178,179,180,181].

One of the clearest clinical settings is IBD. Recent metabolomic studies have shown that fecal and plasma metabolite profiles differ substantially between patients with IBD and controls, and that fecal metabolites in particular can discriminate IBD while also displaying disease-related dietary associations. These data support the idea that stool metabolite profiling may provide a more functional readout of intestinal dysbiosis than microbiome composition alone. Recent reviews and systematic analyses further indicate that recurrent alterations in IBD involve SCFAs, bile acids, amino acid-related metabolites, and microbial co-metabolites, reinforcing their potential diagnostic relevance [180,183,185]. Within these candidate signatures, bile acids appear particularly promising because they may reflect both microbial transformation capacity and disease activity. A 2024 study reported that bile acid profiling could effectively stage pediatric IBD, indicating that defined bile acid patterns may be useful not only for detection but also for stratification of disease severity. This is especially relevant in the present context because disturbed bile acid pools are mechanistically linked to microbial metabolism, epithelial stress, and inflammatory signaling [182]. Biomarker development becomes more compelling when microbiome and metabolome data are integrated rather than analyzed separately. A growing translational literature argues that clinically useful host–microbiota biomarkers in IBD will likely emerge from multi-omic models that combine microbial composition with metabolite measurements and host-response variables, rather than from single analytes alone. In practical terms, the strongest candidates are therefore more likely to be composite signatures, for example. Combinations of SCFA-related readouts, bile acid ratios, aromatic amino acid metabolites, and inflammatory host features [178,186]. A related principle is emerging in colorectal cancer (CRC), where metabolite and epigenetic readouts may be particularly informative when interpreted together. A 2020 multi-omic study identified associations between CRC-related microbial taxa, butyrate-related metabolites, and host DNA methylation-associated gene expression in tumor and normal mucosa, suggesting that metabolite and epigenetic features may jointly improve disease stratification. This type of observation is important because it moves biomarker development beyond exposure measurement alone and toward functional signatures that connect the microbiome to stable host regulatory states [179]. Epigenetic readouts are especially interesting as complementary biomarkers. In IBD, systemic epigenetic alterations have been detected already at diagnosis, supporting the translational potential of methylation-based profiling in complex inflammatory disease. In CRC, the relevance of host DNA methylation as part of microbiome-associated disease biology is even more apparent because methylation changes may reflect longer-lasting host responses to microbial and metabolic cues. Epigenetic features are therefore unlikely to replace metabolite measurements, but they may help distinguish transient exposure from more persistent host reprogramming [179,181]. From a translational perspective, the most informative biomarker strategies will probably be those that integrate microbial composition, metabolite profiles, and host regulatory readouts rather than relying on a single layer alone. This aligns well with the underlying biology of the metabolite–epigenetic–redox axis, in which microbial metabolites are not only present or absent, but are interpreted by host tissues in a context-dependent manner that may leave both metabolic and epigenetic traces. Recent precision-medicine and systems-biology reviews in IBD likewise emphasize that multi-omics integration is likely to be necessary for clinically robust biomarker development [178,186]. However, several limitations still complicate clinical implementation. Metabolite levels are strongly influenced by diet, medication, disease activity, sampling site, and analytical platform, while epigenetic measurements depend heavily on tissue source and cellular composition. Stool, serum, mucosal biopsy material, and isolated immune cells are therefore not interchangeable specimen types. Longitudinal sampling, standardized workflows, and cell type-aware study design will be essential if metabolite and epigenetic signatures are to become robust biomarkers for diagnosis, staging, prognosis, or treatment response [178,186].

Overall, the strongest biomarker potential may lie not in isolated metabolites or single epigenetic features, but in integrated signatures that capture microbial function, host response, and regulatory state at the same time. At present, however, such signatures should be regarded mainly as promising candidates rather than established clinical biomarkers. Such composite approaches are particularly well suited to diseases such as IBD and CRC, in which dysbiosis, metabolite shifts, inflammatory activation, and host chromatin changes occur in parallel and are biologically intertwined [178,179].

### 5.3. Therapeutic Strategies Targeting the Microbiota–Metabolite–Epigenetic Axis

The mechanistic framework outlined above suggests that therapeutic strategies should consider not only the modulation of microbial composition but also the downstream metabolite landscape and the host regulatory machinery through which these signals are interpreted. In practical terms, intervention is possible at several levels, including the modulation of microbial metabolite production through dietary means, the direct administration of metabolites or metabolite mimetics, the manipulation of microbial communities through the use of prebiotics, probiotics, or faecal microbiota transplantation (FMT), and the pharmacological targeting of host redox-sensitive and epigenetic pathways. This broader view is important because clinical efficacy is likely to depend not only on restoring specific metabolite classes, but also on restoring the capacity of host tissues to respond appropriately to these signals (Table 6) [187,188]. However, translation into clinical strategies remains challenging, because much of the mechanistic evidence still comes from experimental models, whereas human studies are often influenced by diet, medication, disease stage, microbiota composition, and host metabolism. Therefore, interventions targeting the microbiota–metabolite–epigenetic axis will require careful validation in well-defined patient cohorts and mechanistically informed clinical studies.

Among microbiota-directed approaches, dietary modulation, prebiotic supplementation, selected probiotic interventions, and FMT represent direct strategies to reshape the intestinal ecosystem and its metabolite output. In active ulcerative colitis, randomized and controlled studies indicate that multidonor FMT can induce clinical and endoscopic improvement, although efficacy depends on donor characteristics, treatment intensity, and patient selection [187,192,193,194,195]. This concept is also being refined in registered studies combining microbiota transfer with dietary modulation, including high-fiber intervention strategies (NCT03998488).

Direct metabolite supplementation has so far yielded a more heterogeneous clinical picture. Butyrate remains mechanistically attractive because it links microbial metabolism to epithelial energy supply, histone deacetylase inhibition, and anti-inflammatory signaling, but its clinical translation has been inconsistent. In children and adolescents with newly diagnosed inflammatory bowel disease, a 12-week adjunctive sodium butyrate regimen did not improve outcomes in one multicenter randomized placebo-controlled trial, whereas a more recent study reported beneficial effects of sodium butyrate supplementation on disease-related clinical and biochemical measures [190,196]. Another ongoing clinical trial is NCT05456763. It follows the same broader translational rationale as the previously published randomized, placebo-controlled, multicenter study of oral sodium butyrate as an add-on therapy for children and adolescents with newly diagnosed inflammatory bowel disease [190]. More broadly, these observations suggest that successful therapeutic modulation of the microbiota–metabolite axis will likely depend on disease context, formulation, dosing, and host responsiveness rather than on a single universal metabolite strategy [190,196,197].

Overall, the available evidence suggests that targeting a single metabolite alone will often not be sufficient. More effective strategies may require a combination of microbiota-directed interventions and modulation of host response pathways. This is particularly relevant for the metabolite–epigenetic–redox axis, since microbial signals are interpreted in the context of chromatin state, inflammatory tone, and redox-sensitive transcriptional programs. In this setting, restoration of microbial metabolite balance may be strengthened by pharmacological targeting of host pathways such as Nrf2 signaling, NF-κB activity, HDACs, DNA methylation, sirtuins, or other disease-relevant chromatin regulators (Table 6) [191,198,199]. Microbiota-directed interventions may help restore the availability of beneficial metabolites, whereas host-directed approaches may restore or enhance the ability of tissues to interpret these signals appropriately. In this context, dimethyl fumarate (DMF) is a useful example. Although it is not a microbiota-derived metabolite, DMF is a clinically used fumarate ester that affects redox-sensitive and stress-related pathways. In cutaneous T cell lymphoma (CTCL), preclinical studies have shown that DMF restores sensitivity to apoptosis and reduces tumor growth and metastasis by inhibiting NF-κB [199]. Subsequent mechanistic studies have also shown that DMF targets thioredoxin-1 and can induce ripoptosome-mediated cell death [200]. These findings were later supported by clinical data: in a multicenter phase II trial involving patients with relapsed or refractory CTCL, DMF demonstrated clinical activity and was well tolerated. This supports further evaluation of combination approaches (NCT02546440) [199]. Taken together, these studies show that a redox-active small molecule can be therapeutically relevant at the interface of inflammatory signaling, oxidative stress adaptation, and tumor cell survival. The translational relevance of such an approach is strengthened further by recent evidence that epigenetic co-targeting can enhance DMF responsiveness. A CRISPR-Cas9 screen identified inhibition of enhancer of zeste homolog 2 (EZH2) as a sensitizer to DMF-induced cell death in malignant T cells, and pharmacologic EZH2 inhibition enhanced the cytotoxic effect of DMF. Although this work was performed in a malignant T cell setting rather than in a microbiota intervention model, it provides clear proof of principle that redox-active metabolic intervention and epigenetic targeting can cooperate therapeutically. In this sense, the study extends the logic of the microbiota–metabolite–epigenetic axis into the translational space by showing that host chromatin regulators can be used to amplify the biological consequences of metabolically relevant stress signaling [191]. The broader significance of such strategies is that they may influence not only inflammation and antioxidant defense, but also cellular stress tolerance, survival thresholds, and susceptibility to regulated cell death. This perspective is particularly relevant in chronic inflammation and cancer, where disease progression may reflect both persistent maladaptive survival signaling and defective elimination of damaged or transformed cells. In that sense, therapeutic targeting of the metabolite–epigenetic–redox axis is conceptually compatible with the more general principle that tissue homeostasis and disease control depend not only on survival pathways, but also on the appropriate execution of cell death programs [191,200].

A major challenge for clinical translation is that responses to microbiota-directed and metabolite-based interventions are likely to vary substantially between patients. Baseline microbiota composition, dietary patterns, medication use, inflammatory activity, and tissue-specific host response programs may all influence whether a given intervention results in a favorable shift in metabolite availability and downstream signaling. This suggests that future therapeutic strategies will likely require better patient stratification and a closer integration of microbiome, metabolite, and host-response profiling in order to identify those individuals most likely to benefit from a given intervention [201,202,203]. At the same time, microbiota-directed and host-directed approaches should not be viewed as competing strategies. This distinction may be particularly important in chronic inflammatory or neoplastic settings, in which both the extracellular metabolite milieu and the intracellular response capacity of host cells are altered [202,203].

Current evidence supports the concept that future therapeutic strategies may need to move beyond simple microbiota correction and instead consider the broader microbiota–metabolite–epigenetic axis. Clinically, this may involve microbiota-directed approaches such as FMT or metabolite supplementation, but it may also require pharmacologic targeting of the host pathways that interpret microbial signals. At this stage, however, such approaches should be viewed as emerging and hypothesis-generating rather than established therapeutic principles. The most promising future strategies are therefore likely to be combination approaches that integrate ecological intervention, metabolite restoration, and host-directed redox or epigenetic modulation, particularly in diseases marked by chronic inflammation, oxidative stress, and maladaptive tissue remodeling [187,188,191,199].

### 5.4. Challenges and Future Perspectives

Despite substantial progress, several challenges still limit a more precise understanding of how microbiota-derived metabolites influence host redox homeostasis through epigenetic mechanisms. A central problem is that many current studies remain associative. In particular, it is often difficult to distinguish whether altered metabolite patterns are primary drivers of disease-related signaling changes or secondary consequences of inflammation, diet, medication, or host metabolic adaptation. In addition, taxonomic, metabolomic, and host-response datasets are often generated in parallel but not mechanistically integrated, which limits causal interpretation [30,178]. Such differences in study design, metabolite dosing, exposure duration, analytical platforms, and disease context likely contribute to inconsistent or divergent findings and should be considered when comparing studies. These limitations also influenced the scope of the present review. As a narrative and mechanistically focused synthesis, it emphasizes mechanistic connections, recurring principles, and context-dependent patterns across metabolite classes rather than providing a systematic inventory of all available studies. In interpreting these findings, we distinguish between mechanisms supported by experimental evidence, associations mainly derived from human observational studies, and emerging hypotheses that require further causal validation. This distinction is particularly important because functional integration of the microbiota–metabolite–epigenetic–redox axis does not necessarily imply that all individual links have been causally demonstrated.

Biological heterogeneity remains another major obstacle. The effects of microbial metabolites depend not only on metabolite identity and concentration, but also on tissue compartment, cell type, inflammatory status, and chromatin configuration. As a result, similar metabolite profiles may lead to different functional outcomes in different disease settings or patient groups. This is particularly relevant for translational studies, where patient stratification will likely be necessary if microbiota-directed or host-directed interventions are to be applied more precisely [178,186,204,205].

Methodological standardization is also still insufficient. Differences in sampling site, biospecimen type, dietary background, analytical platform, and bioinformatic processing continue to limit reproducibility and cross-study comparability. These issues become even more important in multi-omics studies, where variability at one level may affect the interpretation of the entire dataset. More standardized pipelines and better integration of longitudinal sampling with functional validation will therefore be needed [30,178,206].

These limitations also affect clinical translation. Broad approaches such as dietary modulation or fecal microbiota transplantation can be effective in selected settings, but their effects are variable and remain difficult to predict. Recent work indicates that donor and recipient characteristics, microbial diversity, butyrate-producing taxa, and strain-level ecological features all influence engraftment and treatment response. This suggests that future microbiota-based interventions will need to become more defined and mechanistically informed [201,207,208].

A further priority is stronger experimental validation. Omics-based studies can identify candidate metabolite–pathway relationships, but they do not by themselves establish whether a given metabolite directly alters chromatin state, modifies redox control, or contributes to disease progression. Gnotobiotic systems, defined microbial communities, and organoid-based host–microbe models will therefore remain important for testing causality and for resolving tissue-specific and cell-type-specific effects under controlled conditions [209].

Overall, future progress in this field will depend on combining mechanistic studies with standardized multi-omics approaches and clinically meaningful stratification strategies. This will be important not only for clarifying how microbial metabolites regulate epigenetic and redox-sensitive pathways, but also for identifying more robust diagnostic and therapeutic opportunities [178,201].

## 6. Conclusions

Microbiota-derived metabolites have emerged as important regulators of host redox homeostasis and influence cellular responses through mechanisms that extend beyond classical receptor-mediated signaling. As summarized in this review, SCFAs, secondary bile acids, tryptophan-derived metabolites, and other microbiota-modulated compounds affect antioxidant defense, inflammatory signaling, barrier integrity, and immune regulation through partially overlapping pathways. A central aspect of these effects is their capacity to influence chromatin-associated processes, including histone acetylation, DNA methylation, cofactor-dependent epigenetic regulation, and the transcriptional responsiveness of redox-sensitive genes. The available evidence supports the view that epigenetic regulation is not merely a downstream consequence of microbial signaling, but an important mechanistic interface through which microbial metabolites shape host stress adaptation. In this context, redox homeostasis is determined not by a single pathway, but by an integrated network involving Nrf2, NF-κB, AhR, metabolic state, and chromatin configuration. This framework helps explain why similar metabolite classes can exert protective or detrimental effects depending on concentration, cell type, tissue compartment, and disease setting. At the same time, current knowledge remains incomplete. Many studies are still associative, and further work will be needed to clarify causality, improve methodological standardization, and define how microbial metabolite signals are translated into stable host transcriptional programs under physiological and pathological conditions. Overall, the microbiota–metabolite–epigenetic axis provides a useful framework for understanding how microbial metabolism contributes to redox-sensitive host regulation. A better mechanistic understanding of this axis may support the development of more precise biomarkers and more effective therapeutic strategies aimed at restoring redox balance in inflammatory, metabolic, and neoplastic diseases.

## Figures and Tables

**Table 1:** Selected major microbiota-derived and microbiota-modulated metabolite classes and their predominant levels of supporting evidence.

Metabolite Class	Predominant Evidence Base	Main Mechanistic Support	Translational Interpretation
Short-chain fatty acids (SCFAs)	Strong in vitro and animal evidence; supportive human association studies.	HDAC inhibition, GPCR signaling, Nrf2 activation, barrier, and immune regulation.	Most mechanistically established class; clinical relevance supported, but therapeutic use requires context-specific validation.
Secondary bile acids	In vitro and animal evidence; human associations in metabolic, inflammatory, and neoplastic diseases.	FXR, VDR, and TGR5 signaling; mitochondrial stress responses; context-dependent chromatin-associated effects.	Biologically important but highly context-dependent; both protective and deleterious effects need to be considered.
Tryptophan-derived metabolites	In vitro and animal evidence; growing human observational evidence.	AhR-dependent transcriptional regulation, barrier protection, immune modulation, AhR-Nrf2 crosstalk.	Strong conceptual relevance; direct stable epigenetic effects remain less consistently demonstrated.
Polyphenol-derived metabolites	Experimental evidence for selected metabolites; human data are mainly indirect or association-based.	Nrf2 activation, mitochondrial effects, context-dependent HDAC/DNMT modulation.	Promising but heterogeneous; effects depend on microbial conversion, bioavailability, compound identity, and dose.
Hydrogen sulfide	Strong mechanistic evidence in experimental systems; limited direct human validation.	Persulfidation, Keap1/Nrf2 activation, mitochondrial effects depending on concentration.	Dual protective or deleterious effects; translational relevance depends on local concentration and source.
Lipid mediators	Experimental and clinical association evidence; microbiota influence is often indirect.	Inflammatory resolution, Nrf2/NF-κB regulation, mitochondrial ROS modulation, chromatin-associated inflammatory programs.	Relevant to inflammation and resolution, but often microbiota-modulated rather than directly microbiota-derived.

This table summarizes the predominant evidence supporting each metabolite class. Evidence for extraintestinal effects varies between metabolites and tissues; therefore, the translational interpretation reflects the current strength and specificity of the available data. Abbreviations: AhR, aryl hydrocarbon receptor; DNMT, DNA methyltransferase; FXR, farnesoid X receptor; GPCR, G-protein-coupled receptor; HDAC, histone deacetylase; NF-κB, nuclear factor kappa-light-chain-enhancer of activated B cells; Nrf2, nuclear factor erythroid 2-related factor 2; ROS, reactive oxygen species; TGR5, G-protein-coupled bile acid receptor 1; VDR, vitamin D receptor.

**Table 2:** Selected epigenetic enzyme families, their functions, metabolic dependencies, mechanisms of redox sensitivity, and relevance for antioxidant and stress-responsive gene regulation.

Enzyme/Enzyme Family	Epigenetic Function	Key Cofactors or Metabolic Dependencies	Redox-Sensitive Regulatory Mechanism	Major Consequences for Gene Regulation	Relevance to Redox Homeostasis
Class I HDACs (HDAC1/2/3) [130,131]	Histone deacetylation; transcriptional repression	Zn^2+^-dependent deacetylases	Inactivation by electrophilic lipid peroxidation products such as 4-HNE and 15d-PGJ2 via modification of conserved cysteine residues.	Derepression of stress-responsive and cytoprotective genes, including HO-1 and HSP70.	Links oxidative stress directly to chromatin relaxation and induction of defense pathways.
Class III HDACs (Sirtuins) [128,129]	NAD^+^-dependent deacetylation of histones and non-histone proteins	NAD^+^; NAD^+^/NADH ratio	Activity changes with cellular energy and redox state.	Alters stress adaptation, mitochondrial programs, inflammatory signaling, and antioxidant responses.	Couples metabolic/redox status to transcriptional regulation.
HATs (e.g., p300/CBP-containing complexes) [129,130]	Histone acetylation; chromatin opening	Acetyl-CoA	Indirectly modulated by oxidative/inflammatory signaling and metabolite availability.	Increased transcription of accessible chromatin regions, including inflammatory and stress-response genes.	Supports rapid transcriptional responses during oxidative and inflammatory stress.
DNMTs [128,132]	DNA methylation	SAM	Sensitive to redox-dependent changes in methyl donor availability.	Changes in methylation capacity may affect long-term gene repression programs.	Connects glutathione/redox balance with stable transcriptional regulation.
TET1/2/3 [133,134,135]	Active DNA demethylation via oxidation of 5mC	Fe(II), 2-OG, O_2_, ascorbate	Inhibited by oxidation of Fe(II); influenced by oxygen availability and metabolite competition by succinate/fumarate.	Alters DNA methylation landscapes and expression of differentiation-, antioxidant-, and adaptation-related genes.	Central redox-sensitive node linking metabolism to DNA demethylation.
JmjC histone demethylases [132,136]	Histone lysine demethylation	Fe(II), 2-OG, O_2_	Activity reduced by oxidative stress; sensitive to hypoxia and metabolite competition.	Changes histone methylation states and chromatin accessibility at stress-relevant loci.	Integrates oxygen, iron redox state, and metabolism with chromatin regulation.
					data
					data
KDM2B/Ndy1 [137]	Histone demethylation at specific loci	Fe(II), 2-OG	Redox-sensitive demethylase with functional impact on antioxidant gene expression.	Promotes transcription of antioxidant genes such as NQO1, PRDX4, and AASS.	Example of epigenetic enzymes actively shaping redox homeostasis.
PARPs [128,129]	Poly-ADP-ribosylation; chromatin-associated stress responses	NAD^+^	Activated in stress contexts and coupled to NAD^+^ metabolism.	Affects chromatin remodeling, DNA damage responses, and transcriptional control.	Links redox stress, energy metabolism, and chromatin function.

Abbreviations: 2-OG, 2-oxoglutarate; AASS, aminoadipate-semialdehyde synthase; DNMT, DNA methyltransferase; Fe(II), ferrous iron; HAT, histone acetyltransferase; HDAC, histone deacetylase; HSP70, heat shock protein 70; JmjC, Jumonji C domain-containing; KDM2B, lysine demethylase 2B; NAD^+^, nicotinamide adenine dinucleotide; NQO1, NAD(P)H quinone dehydrogenase 1; PARP, poly(ADP-ribose) polymerase; PRDX4, peroxiredoxin 4; SAM, S-adenosylmethionine; TET, ten-eleven translocation.

**Table 3:** Selected redox-sensitive epigenetic enzyme families linking metabolic state, chromatin regulation, and stress-responsive gene expression.

Histone Modification	Biochemical Source/Donor	Association with Chromatin State	Connection to Redox Biology	Functional Relevance	Current Level of Evidence
Histone acetylation	Acetyl-CoA	Generally associated with open chromatin and active transcription.	Sensitive to oxidative effects on HATs/HDACs and acetyl-CoA availability.	Regulates inflammatory, metabolic, and stress-response genes.	Strong [130,132,142]
Histone crotonylation	Crotonyl-CoA	Often associated with active promoters and enhancers.	Links short-chain acyl metabolism to transcriptional regulation.	May regulate metabolism- and stress-associated gene programs.	Moderate to strong [143]
Histone lactylation	Lactate-derived donor pools	Associated with transcriptionally active states in selected contexts.	Connects glycolytic/inflammatory metabolism to chromatin regulation.	Implicated in immune and stress-related transcriptional responses.	Emerging [138]
Histone glutathionylation	Glutathione	May alter histone-DNA interactions and chromatin behavior.	Directly reflects redox environment and glutathione status.	Links oxidative stress buffering to chromatin structure.	Emerging [138]
Histone carbonylation	Reactive carbonyl species	Often associated with oxidative damage-related chromatin changes.	Direct consequence of oxidative/electrophilic stress.	May disturb chromatin organization and protein interactions.	Emerging [138]
Histone nitrosylation	Reactive nitrogen species	Context-dependent.	Connects nitrosative stress to chromatin regulation.	Potentially alters transcriptional control under inflammatory stress.	Emerging [138]
Histone methylation changes under oxidative stress	SAM-dependent methylation machinery	Context-dependent; may accumulate when demethylases are inhibited.	Linked indirectly to oxidative inhibition of JmjC demethylases.	Alters gene expression programs involved in adaptation or pathology.	Moderate [132,138]

Abbreviations: CoA, coenzyme A; DNA, deoxyribonucleic acid; HAT, histone acetyltransferase; HDAC, histone deacetylase; JmjC, Jumonji C domain-containing; SAM, S-adenosylmethionine.

**Table 4:** Selected metabolites and cofactors that act as substrates, cofactors, inhibitors, or biochemical constraints for epigenetic enzymes and thereby connect metabolic state to chromatin regulation.

Metabolite/Cofactor	Epigenetic Processes Influenced	Main Target Enzymes or Pathways	Effect of Altered Redox/Metabolic State	Functional Consequences	Potential Relevance to Microbiota-Derived Metabolism	Key References
NAD^+^	Histone deacetylation; ADP-ribosylation	Sirtuins, PARPs	Altered NAD^+^/NADH ratio changes enzyme activity.	Affects stress adaptation, mitochondrial function, and inflammatory signaling.	Can be influenced indirectly through microbial modulation of host metabolism and tryptophan–kynurenine–NAD^+^ pathways.	[128,129]
SAM	DNA and histone methylation	DNMTs, histone methyltransferases	Oxidative/glutathione-dependent limitation of SAM synthesis may reduce methylation capacity.	Alters long-term transcriptional repression and epigenetic stability.	May be indirectly shaped by microbiota-dependent effects on one-carbon and sulfur metabolism.	[128,138]
2-OG (α-ketoglutarate)	DNA and histone demethylation	TET enzymes, JmjC demethylases	Reduced availability limits dioxygenase activity.	Affects chromatin plasticity, antioxidant gene regulation, and adaptation programs.	Represents a key metabolic node potentially influenced by microbial effects on host intermediary metabolism.	[129,135,136]
Succinate	Inhibits demethylation reactions	TET enzymes, JmjC demethylases	Accumulation antagonizes 2-OG-dependent dioxygenases.	Favors hypermethylation-like states and reduced demethylation capacity.	May reflect host metabolic states shaped by microbial metabolites.	[134,135]
Fumarate	Inhibits demethylation reactions	TET enzymes, JmjC demethylases	Accumulation restrains dioxygenase activity.	Promotes altered chromatin regulation and persistent stress-associated gene expression	Relevant as a metabolic intermediate and as a conceptual link to electrophilic stress biology.	[134,135]
Acetyl-CoA	Histone acetylation	HATs	Availability determines substrate pool for acetylation.	Influences chromatin accessibility and transcriptional activation.	Can be connected to SCFA metabolism, especially acetate and butyrate-related carbon flux.	[134,135]
Crotonyl-CoA	Histone crotonylation	Histone acylation pathways	Availability may affect crotonylation levels.	Associated with active promoters/enhancers and stress-related transcription.	Potentially linked to SCFA-associated acyl metabolism.	[143]
Lactate	Histone lactylation	Lactylation-associated chromatin pathways	Accumulates under glycolytic/inflammatory conditions.	May influence immune and stress-response transcriptional programs.	Microbiota may contribute indirectly through metabolic reprogramming and lactate cross-feeding.	[138]
Ascorbate	Supports demethylation reactions	TET enzymes; possibly JmjC enzymes	Maintains Fe(II) state and supports dioxygenase activity.	Promotes DNA/histone demethylation capacity.	Not microbiota-derived, but relevant as a host cofactor shaping metabolite-sensitive epigenetic responses.	[136]
Glutathione (GSH/GSSG)	Indirect regulation of methylation and redox-associated PTMs	MAT1A/SAM pathway; redox-sensitive chromatin context	Oxidative shift may limit methyl donor availability and alter redox-sensitive enzyme function.	Links antioxidant buffering capacity to epigenetic stability.	Important host redox buffer affected indirectly by microbial metabolites and inflammation.	[128,138]

Abbreviations: 2-OG, 2-oxoglutarate; CoA, coenzyme A; DNMT, DNA methyltransferase; DNA, deoxyribonucleic acid; Fe(II), ferrous iron; GSH, reduced glutathione; GSSG, oxidized glutathione; HAT, histone acetyltransferase; JmjC, Jumonji C domain-containing; MAT1A, methionine adenosyltransferase 1A; NAD^+^, nicotinamide adenine dinucleotide; NADH, reduced nicotinamide adenine dinucleotide; PARP, poly(ADP-ribose) polymerase; PTMs, post-translational modifications; SAM, S-adenosylmethionine; SCFA, short-chain fatty acids; TET, ten-eleven translocation.

**Table 5:** Candidate metabolite and epigenetic signatures in inflammatory and neoplastic disease contexts.

Disease Context	Candidate Signature	Sample Type	Potential Biomarker Application	Main Limitations	References
Inflammatory bowel disease (IBD)	Global fecal/plasma metabolite shifts; recurrent alterations in SCFAs, bile acids, amino acid-related metabolites, and microbial co-metabolites	Stool, plasma	Disease discrimination; functional readout of dysbiosis.	Strong dependence on diet, medication, disease activity, and sampling compartment.	[180]
Pediatric IBD	Bile acid profiling	Serum/plasma	Disease staging and severity stratification.	Age dependence; bile acid pools are influenced by diet and intestinal transit.	[182]
IBD (multi-omics/discovery setting)	Composite metabolite signatures integrating SCFA-related readouts, bile acids, amino acid metabolites, and microbial co-metabolites	Primarily stool; sometimes plasma	Biomarker discovery; patient stratification; pathway-level classification.	Composite signatures may be cohort- and platform-specific.	[183,184]
Colorectal cancer (CRC)	Associations between CRC-related microbial taxa, butyrate-related metabolites, and host DNA methylation-associated gene expression	Tumor tissue, adjacent mucosa, microbiome/metabolome profiles	Disease stratification; mechanistic subclassification; integrated host–microbiota biomarker model.	Tissue specificity and cohort heterogeneity may limit direct clinical translation.	[179]
IBD	Systemic epigenetic alterations detectable already at diagnosis	Peripheral blood	Diagnosis; risk stratification; potential prediction of treatment escalation.	Highly cell type-dependent; methylation signatures may not be disease-specific in isolation.	[181]
IBD/translational biomarker setting	Integrated microbiome–metabolome–host-response signatures	Stool, plasma, blood, mucosal tissue	Diagnosis, staging, prognosis, treatment response monitoring.	Requires standardized multi-omics workflows and longitudinal validation.	[181,183]

Abbreviations: CRC, colorectal cancer; DNA, deoxyribonucleic acid; IBD, inflammatory bowel disease; SCFAs, short-chain fatty acids.

**Table 6:** Selected therapeutic strategies targeting the microbiota–metabolite–epigenetic axis.

Therapeutic Approach	Example/Intervention	Disease Context	Outcome and Proposed Mechanism	Evidence Level	References/Clinical Trial
Microbiota-directed ecosystem modulation	Multidonor FMT	Active ulcerative colitis	Can induce clinical and endoscopic improvement in active ulcerative colitis; proposed mechanism includes reshaping microbial community structure and downstream metabolite output, with restoration of microbial functions linked to barrier integrity and inflammatory control.	Randomized controlled trials; meta-analyses	[187,189]/NCT03998488
Dietary modulation	High-fiber intervention strategies	Ulcerative colitis/microbiota-targeted intervention studies	Therapeutic efficacy is being evaluated in microbiota-targeted intervention studies; proposed mechanism includes altered substrate availability for microbial fermentation, promotion of short-chain fatty acid production, and broader beneficial metabolite shifts.	Clinical study designs/registered trials	NCT03998488
Direct metabolite supplementation	Oral sodium butyrate	Pediatric inflammatory bowel disease	Clinical data are heterogeneous, with no clear benefit in one randomized placebo-controlled trial but beneficial clinical and biochemical effects reported in another study; proposed mechanism includes support of epithelial energy metabolism, HDAC-related effects, barrier function, and inflammatory regulation.	Mixed clinical data; ongoing trial	[190]/NCT05456763
Epigenetic co-targeting	EZH2 inhibition + DMF	T cell lymphoma	Shows preclinical synergistic activity by enhancing susceptibility to DMF-induced cell death; proposed mechanism involves combined redox-active and chromatin-directed targeting through EZH2 inhibition and DMF-mediated stress pathway modulation	Preclinical CRISPR-Cas9 and pharmacologic study	[191]

This table presents selected representative examples and is not intended to provide a systematic or exhaustive overview of all therapeutic studies in this field. Abbreviations: DMF, dimethyl fumarate; EZH2, enhancer of zeste homolog 2; FMT, fecal microbiota transplantation; HDAC, histone deacetylase; NCT, ClinicalTrials.gov identifier.

## Data Availability

No new data were created or analyzed in this study. Data sharing is not applicable to this article.

## References

[1] Lynch S.V., Pedersen O. (2016). The Human Intestinal Microbiome in Health and Disease. N. Engl. J. Med..

[2] Levy M., Kolodziejczyk A.A., Thaiss C.A., Elinav E. (2017). Dysbiosis and the immune system. Nat. Rev. Immunol..

[3] Chidambaram S.B., Essa M.M., Rathipriya A.G., Bishir M., Ray B., Mahalakshmi A.M., Tousif A.H., Sakharkar M.K., Kashyap R.S., Friedland R.P. (2022). Gut dysbiosis, defective autophagy, and altered immune responses in neurodegenerative diseases: Tales of a vicious cycle. Pharmacol. Ther..

[4] Agus A., Clement K., Sokol H. (2021). Gut microbiota-derived metabolites as central regulators in metabolic disorders. Gut.

[5] Woo V., Alenghat T. (2022). Epigenetic regulation by gut microbiota. Gut Microbes.

[6] Pizzino G., Irrera N., Cucinotta M., Pallio G., Mannino F., Arcoraci V., Squadrito F., Altavilla D., Bitto A. (2017). Oxidative Stress: Harms and Benefits for Human Health. Oxid. Med. Cell Longev..

[7] Gonzalez-Bosch C., Boorman E., Zunszain P.A., Mann G.E. (2021). Short-chain fatty acids as modulators of redox signaling in health and disease. Redox Biol..

[8] Hayes J.D., Dinkova-Kostova A.T. (2014). The Nrf2 regulatory network provides an interface between redox and intermediary metabolism. Trends Biochem. Sci..

[9] Seitz R., Tumen D., Kunst C., Heumann P., Schmid S., Kandulski A., Muller M., Gulow K. (2024). Exploring the Thioredoxin System as a Therapeutic Target in Cancer: Mechanisms and Implications. Antioxidants.

[10] Seitz R., Muller M., Gulow K. (2026). Extracellular Redox Balance as a Determinant of Immune Regulation and Tissue Inflammation. Antioxidants.

[11] Munteanu C., Galaction A.I., Turnea M., Blendea C.D., Rotariu M., Postaru M. (2024). Redox Homeostasis, Gut Microbiota, and Epigenetics in Neurodegenerative Diseases: A Systematic Review. Antioxidants.

[12] Mukhopadhya I., Louis P. (2025). Gut microbiota-derived short-chain fatty acids and their role in human health and disease. Nat. Rev. Microbiol..

[13] Mann E.R., Lam Y.K., Uhlig H.H. (2024). Short-chain fatty acids: Linking diet, the microbiome and immunity. Nat. Rev. Immunol..

[14] Masse K.E., Lu V.B. (2023). Short-chain fatty acids, secondary bile acids and indoles: Gut microbial metabolites with effects on enteroendocrine cell function and their potential as therapies for metabolic disease. Front. Endocrinol..

[15] Tan Y.Q., Wang Y.N., Feng H.Y., Guo Z.Y., Li X., Nie X.L., Zhao Y.Y. (2022). Host/microbiota interactions-derived tryptophan metabolites modulate oxidative stress and inflammation via aryl hydrocarbon receptor signaling. Free Radic. Biol. Med..

[16] Ho R.H., Chan J.C.Y., Fan H., Kioh D.Y.Q., Lee B.W., Chan E.C.Y. (2017). In Silico and in Vitro Interactions between Short Chain Fatty Acids and Human Histone Deacetylases. Biochemistry.

[17] Thomas S.P., Denu J.M. (2021). Short-chain fatty acids activate acetyltransferase p300. eLife.

[18] Kunst C., Schmid S., Michalski M., Tumen D., Buttenschon J., Muller M., Gulow K. (2023). The Influence of Gut Microbiota on Oxidative Stress and the Immune System. Biomedicines.

[19] Lund P.J., Gates L.A., Leboeuf M., Smith S.A., Chau L., Lopes M., Friedman E.S., Saiman Y., Kim M.S., Shoffler C.A. (2022). Stable isotope tracing in vivo reveals a metabolic bridge linking the microbiota to host histone acetylation. Cell Rep..

[20] Ferreyra J.A., Wu K.J., Hryckowian A.J., Bouley D.M., Weimer B.C., Sonnenburg J.L. (2014). Gut microbiota-produced succinate promotes C. difficile infection after antibiotic treatment or motility disturbance. Cell Host Microbe.

[21] Ghosh S., Dai C., Brown K., Rajendiran E., Makarenko S., Baker J., Ma C., Halder S., Montero M., Ionescu V.A. (2011). Colonic microbiota alters host susceptibility to infectious colitis by modulating inflammation, redox status, and ion transporter gene expression. Am. J. Physiol. Gastrointest. Liver Physiol..

[22] Ng K.M., Ferreyra J.A., Higginbottom S.K., Lynch J.B., Kashyap P.C., Gopinath S., Naidu N., Choudhury B., Weimer B.C., Monack D.M. (2013). Microbiota-liberated host sugars facilitate post-antibiotic expansion of enteric pathogens. Nature.

[23] Honda K., Littman D.R. (2016). The microbiota in adaptive immune homeostasis and disease. Nature.

[24] Kaiko G.E., Ryu S.H., Koues O.I., Collins P.L., Solnica-Krezel L., Pearce E.J., Pearce E.L., Oltz E.M., Stappenbeck T.S. (2016). The Colonic Crypt Protects Stem Cells from Microbiota-Derived Metabolites. Cell.

[25] Kelly C.J., Zheng L., Campbell E.L., Saeedi B., Scholz C.C., Bayless A.J., Wilson K.E., Glover L.E., Kominsky D.J., Magnuson A. (2015). Crosstalk between Microbiota-Derived Short-Chain Fatty Acids and Intestinal Epithelial HIF Augments Tissue Barrier Function. Cell Host Microbe.

[26] Rowland I., Gibson G., Heinken A., Scott K., Swann J., Thiele I., Tuohy K. (2018). Gut microbiota functions: Metabolism of nutrients and other food components. Eur. J. Nutr..

[27] Klepsch V., Moschen A.R., Tilg H., Baier G., Hermann-Kleiter N. (2019). Nuclear Receptors Regulate Intestinal Inflammation in the Context of IBD. Front. Immunol..

[28] Wang J., Zhu N., Su X., Gao Y., Yang R. (2023). Gut-Microbiota-Derived Metabolites Maintain Gut and Systemic Immune Homeostasis. Cells.

[29] Schroeder B.O., Backhed F. (2016). Signals from the gut microbiota to distant organs in physiology and disease. Nat. Med..

[30] Kim S., Seo S.U., Kweon M.N. (2024). Gut microbiota-derived metabolites tune host homeostasis fate. Semin. Immunopathol..

[31] Koh A., De Vadder F., Kovatcheva-Datchary P., Backhed F. (2016). From Dietary Fiber to Host Physiology: Short-Chain Fatty Acids as Key Bacterial Metabolites. Cell.

[32] Morrison D.J., Preston T. (2016). Formation of short chain fatty acids by the gut microbiota and their impact on human metabolism. Gut Microbes.

[33] Abdelhalim K.A. (2024). Short-chain fatty acids (SCFAs) from gastrointestinal disorders, metabolism, epigenetics, central nervous system to cancer—A mini-review. Chem. Biol. Interact..

[34] Tan J.K., Macia L., Mackay C.R. (2023). Dietary fiber and SCFAs in the regulation of mucosal immunity. J. Allergy Clin. Immunol..

[35] Campos-Perez W., Martinez-Lopez E. (2021). Effects of short chain fatty acids on metabolic and inflammatory processes in human health. Biochim. Biophys. Acta Mol. Cell Biol. Lipids.

[36] Parada Venegas D., De la Fuente M.K., Landskron G., Gonzalez M.J., Quera R., Dijkstra G., Harmsen H.J.M., Faber K.N., Hermoso M.A. (2019). Corrigendum: Short Chain Fatty Acids (SCFAs)-Mediated Gut Epithelial and Immune Regulation and Its Relevance for Inflammatory Bowel Diseases. Front. Immunol..

[37] Tan J., McKenzie C., Potamitis M., Thorburn A.N., Mackay C.R., Macia L. (2014). The role of short-chain fatty acids in health and disease. Adv. Immunol..

[38] Zhao Y., Chen J., Qin Y., Yuan J., Yu Z., Ma R., Liu F., Zhao J. (2025). Linking Short-Chain Fatty Acids to Systemic Homeostasis: Mechanisms, Therapeutic Potential, and Future Directions. J. Nutr. Metab..

[39] Liu X.F., Shao J.H., Liao Y.T., Wang L.N., Jia Y., Dong P.J., Liu Z.Z., He D.D., Li C., Zhang X. (2023). Regulation of short-chain fatty acids in the immune system. Front. Immunol..

[40] Arpaia N., Campbell C., Fan X., Dikiy S., van der Veeken J., deRoos P., Liu H., Cross J.R., Pfeffer K., Coffer P.J. (2013). Metabolites produced by commensal bacteria promote peripheral regulatory T-cell generation. Nature.

[41] Davie J.R. (2003). Inhibition of histone deacetylase activity by butyrate. J. Nutr..

[42] Huang J., Wang L., Wang S., Lu Y., Zhang W., Wang J. (2018). Spatial and temporal characteristics of temperature effects on cardiovascular disease in Southern China using the Empirical Mode Decomposition method. Sci. Rep..

[43] Furusawa Y., Obata Y., Fukuda S., Endo T.A., Nakato G., Takahashi D., Nakanishi Y., Uetake C., Kato K., Kato T. (2013). Commensal microbe-derived butyrate induces the differentiation of colonic regulatory T cells. Nature.

[44] Hao F., Tian M., Zhang X., Jin X., Jiang Y., Sun X., Wang Y., Peng P., Liu J., Xia C. (2021). Butyrate enhances CPT1A activity to promote fatty acid oxidation and iTreg differentiation. Proc. Natl. Acad. Sci. USA.

[45] Sabari B.R., Tang Z., Huang H., Yong-Gonzalez V., Molina H., Kong H.E., Dai L., Shimada M., Cross J.R., Zhao Y. (2015). Intracellular crotonyl-CoA stimulates transcription through p300-catalyzed histone crotonylation. Mol. Cell.

[46] Hamer H.M., Jonkers D., Venema K., Vanhoutvin S., Troost F.J., Brummer R.J. (2008). Review article: The role of butyrate on colonic function. Aliment. Pharmacol. Ther..

[47] Saeedi B.J., Liu K.H., Owens J.A., Hunter-Chang S., Camacho M.C., Eboka R.U., Chandrasekharan B., Baker N.F., Darby T.M., Robinson B.S. (2020). Gut-Resident Lactobacilli Activate Hepatic Nrf2 and Protect Against Oxidative Liver Injury. Cell Metab..

[48] Vinolo M.A., Rodrigues H.G., Nachbar R.T., Curi R. (2011). Regulation of inflammation by short chain fatty acids. Nutrients.

[49] Scheppach W. (1994). Effects of short chain fatty acids on gut morphology and function. Gut.

[50] Canani R.B., Costanzo M.D., Leone L., Pedata M., Meli R., Calignano A. (2011). Potential beneficial effects of butyrate in intestinal and extraintestinal diseases. World J. Gastroenterol..

[51] Zhang Y., Zhang J., Duan L. (2022). The role of microbiota-mitochondria crosstalk in pathogenesis and therapy of intestinal diseases. Pharmacol. Res..

[52] Kespohl M., Vachharajani N., Luu M., Harb H., Pautz S., Wolff S., Sillner N., Walker A., Schmitt-Kopplin P., Boettger T. (2017). The Microbial Metabolite Butyrate Induces Expression of Th1-Associated Factors in CD4(+) T Cells. Front. Immunol..

[53] Park J., Kim M., Kang S.G., Jannasch A.H., Cooper B., Patterson J., Kim C.H. (2015). Short-chain fatty acids induce both effector and regulatory T cells by suppression of histone deacetylases and regulation of the mTOR-S6K pathway. Mucosal Immunol..

[54] Nshanian M., Gruber J.J., Geller B.S., Chleilat F., Lancaster S.M., White S.M., Alexandrova L., Camarillo J.M., Kelleher N.L., Zhao Y. (2025). Short-chain fatty acid metabolites propionate and butyrate are unique epigenetic regulatory elements linking diet, metabolism and gene expression. Nat. Metab..

[55] Cox A.J., West N.P., Cripps A.W. (2015). Obesity, inflammation, and the gut microbiota. Lancet Diabetes Endocrinol..

[56] Brown A.J., Goldsworthy S.M., Barnes A.A., Eilert M.M., Tcheang L., Daniels D., Muir A.I., Wigglesworth M.J., Kinghorn I., Fraser N.J. (2003). The Orphan G protein-coupled receptors GPR41 and GPR43 are activated by propionate and other short chain carboxylic acids. J. Biol. Chem..

[57] Kim M.H., Kang S.G., Park J.H., Yanagisawa M., Kim C.H. (2013). Short-chain fatty acids activate GPR41 and GPR43 on intestinal epithelial cells to promote inflammatory responses in mice. Gastroenterology.

[58] Biagioli M., Marchiano S., Di Giorgio C., Rondini E., Urbani G., Distrutti E., Fiorucci S. (2025). Secondary bile acids and host metabolism: Crosstalk, signaling pathways and therapeutic frontiers. Eur. Rev. Med. Pharmacol. Sci..

[59] Wahlstrom A., Sayin S.I., Marschall H.U., Backhed F. (2016). Intestinal Crosstalk between Bile Acids and Microbiota and Its Impact on Host Metabolism. Cell Metab..

[60] Fiorucci S., Biagioli M., Zampella A., Distrutti E. (2018). Bile Acids Activated Receptors Regulate Innate Immunity. Front. Immunol..

[61] Jia W., Xie G., Jia W. (2018). Bile acid-microbiota crosstalk in gastrointestinal inflammation and carcinogenesis. Nat. Rev. Gastroenterol. Hepatol..

[62] Sabit H., Cevik E., Tombuloglu H. (2019). Colorectal cancer: The epigenetic role of microbiome. World J. Clin. Cases.

[63] Gadaleta R.M., Oldenburg B., Willemsen E.C., Spit M., Murzilli S., Salvatore L., Klomp L.W., Siersema P.D., van Erpecum K.J., van Mil S.W. (2011). Activation of bile salt nuclear receptor FXR is repressed by pro-inflammatory cytokines activating NF-kappaB signaling in the intestine. Biochim. Biophys. Acta.

[64] Makishima M., Lu T.T., Xie W., Whitfield G.K., Domoto H., Evans R.M., Haussler M.R., Mangelsdorf D.J. (2002). Vitamin D receptor as an intestinal bile acid sensor. Science.

[65] Bernstein H., Bernstein C. (2023). Bile acids as carcinogens in the colon and at other sites in the gastrointestinal system. Exp. Biol. Med..

[66] Fleishman J.S., Kumar S. (2024). Bile acid metabolism and signaling in health and disease: Molecular mechanisms and therapeutic targets. Signal Transduct. Target. Ther..

[67] Payne C.M., Weber C., Crowley-Skillicorn C., Dvorak K., Bernstein H., Bernstein C., Holubec H., Dvorakova B., Garewal H. (2007). Deoxycholate induces mitochondrial oxidative stress and activates NF-kappaB through multiple mechanisms in HCT-116 colon epithelial cells. Carcinogenesis.

[68] Pols T.W., Noriega L.G., Nomura M., Auwerx J., Schoonjans K. (2011). The bile acid membrane receptor TGR5 as an emerging target in metabolism and inflammation. J. Hepatol..

[69] Chiang J.Y.L., Ferrell J.M. (2020). Bile acid receptors FXR and TGR5 signaling in fatty liver diseases and therapy. Am. J. Physiol. Gastrointest. Liver Physiol..

[70] Shin D.J., Wang L. (2019). Bile Acid-Activated Receptors: A Review on FXR and Other Nuclear Receptors. Handb. Exp. Pharmacol..

[71] Belka M., Gostynska-Stawna A., Stawny M., Krajka-Kuzniak V. (2024). Activation of Nrf2 and FXR via Natural Compounds in Liver Inflammatory Disease. Int. J. Mol. Sci..

[72] Wang N., Zou Q., Xu J., Zhang J., Liu J. (2018). Ligand binding and heterodimerization with retinoid X receptor alpha (RXRalpha) induce farnesoid X receptor (FXR) conformational changes affecting coactivator binding. J. Biol. Chem..

[73] Rizzo G., Renga B., Antonelli E., Passeri D., Pellicciari R., Fiorucci S. (2005). The methyl transferase PRMT1 functions as co-activator of farnesoid X receptor (FXR)/9-cis retinoid X receptor and regulates transcription of FXR responsive genes. Mol. Pharmacol..

[74] Parks D.J., Blanchard S.G., Bledsoe R.K., Chandra G., Consler T.G., Kliewer S.A., Stimmel J.B., Willson T.M., Zavacki A.M., Moore D.D. (1999). Bile acids: Natural ligands for an orphan nuclear receptor. Science.

[75] Bramlett K.S., Yao S., Burris T.P. (2000). Correlation of farnesoid X receptor coactivator recruitment and cholesterol 7alpha-hydroxylase gene repression by bile acids. Mol. Genet. Metab..

[76] Yan W., Zhang K., Guo J., Xu L. (2025). Bile acid-mediated gut-liver axis crosstalk: The role of nuclear receptor signaling in dynamic regulation of inflammatory networks. Front. Immunol..

[77] Wang Y.D., Chen W.D., Wang M., Yu D., Forman B.M., Huang W. (2008). Farnesoid X receptor antagonizes nuclear factor kappaB in hepatic inflammatory response. Hepatology.

[78] Seok S., Kanamaluru D., Xiao Z., Ryerson D., Choi S.E., Suino-Powell K., Xu H.E., Veenstra T.D., Kemper J.K. (2013). Bile acid signal-induced phosphorylation of small heterodimer partner by protein kinase Czeta is critical for epigenomic regulation of liver metabolic genes. J. Biol. Chem..

[79] Allen K., Jaeschke H., Copple B.L. (2011). Bile acids induce inflammatory genes in hepatocytes: A novel mechanism of inflammation during obstructive cholestasis. Am. J. Pathol..

[80] Leung H., Xiong L., Ni Y., Busch A., Bauer M., Press A.T., Panagiotou G. (2023). Impaired flux of bile acids from the liver to the gut reveals microbiome-immune interactions associated with liver damage. npj Biofilms Microbiomes.

[81] Yang S.F., Chen X.C., Pan Y.J. (2025). Microbiota-derived metabolites in tumorigenesis: Mechanistic insights and therapeutic implications. Front. Pharmacol..

[82] Wang X., Luo X., Xiao R., Liu X., Zhou F., Jiang D., Bai J., Cui M., You L., Zhao Y. (2026). Targeting metabolic-epigenetic-immune axis in cancer: Molecular mechanisms and therapeutic implications. Signal Transduct. Target. Ther..

[83] Xue C., Li G., Zheng Q., Gu X., Shi Q., Su Y., Chu Q., Yuan X., Bao Z., Lu J. (2023). Tryptophan metabolism in health and disease. Cell Metab..

[84] Roager H.M., Licht T.R. (2018). Microbial tryptophan catabolites in health and disease. Nat. Commun..

[85] Zelante T., Iannitti R.G., Cunha C., De Luca A., Giovannini G., Pieraccini G., Zecchi R., D’Angelo C., Massi-Benedetti C., Fallarino F. (2013). Tryptophan catabolites from microbiota engage aryl hydrocarbon receptor and balance mucosal reactivity via interleukin-22. Immunity.

[86] Scott S.A., Fu J., Chang P.V. (2020). Microbial tryptophan metabolites regulate gut barrier function via the aryl hydrocarbon receptor. Proc. Natl. Acad. Sci. USA.

[87] Vyhlidalova B., Krasulova K., Pecinkova P., Marcalikova A., Vrzal R., Zemankova L., Vanco J., Travnicek Z., Vondracek J., Karasova M. (2020). Gut Microbial Catabolites of Tryptophan Are Ligands and Agonists of the Aryl Hydrocarbon Receptor: A Detailed Characterization. Int. J. Mol. Sci..

[88] Opitz C.A., Holfelder P., Prentzell M.T., Trump S. (2023). The complex biology of aryl hydrocarbon receptor activation in cancer and beyond. Biochem. Pharmacol..

[89] Hou J.J., Ma A.H., Qin Y.H. (2023). Activation of the aryl hydrocarbon receptor in inflammatory bowel disease: Insights from gut microbiota. Front. Cell Infect. Microbiol..

[90] Coumoul X., Barouki R., Esser C., Haarmann-Stemmann T., Lawrence B.P., Lehmann J., Moura-Alves P., Murray I.A., Opitz C.A., Perdew G.H. (2026). The aryl hydrocarbon receptor: Structure, signaling, physiology and pathology. Signal Transduct. Target. Ther..

[91] Schnekenburger M., Peng L., Puga A. (2007). HDAC1 bound to the Cyp1a1 promoter blocks histone acetylation associated with Ah receptor-mediated trans-activation. Biochim. Biophys. Acta.

[92] Habano W., Miura T., Terashima J., Ozawa S. (2022). Aryl hydrocarbon receptor as a DNA methylation reader in the stress response pathway. Toxicology.

[93] Quintana F.J., Basso A.S., Iglesias A.H., Korn T., Farez M.F., Bettelli E., Caccamo M., Oukka M., Weiner H.L. (2008). Control of T(reg) and T(H)17 cell differentiation by the aryl hydrocarbon receptor. Nature.

[94] Stojanovic B., Milivojcevic Bevc I., Dimitrijevic Stojanovic M., Stojanovic B.S., Jovanovic M., Lazarevic S., Milosevic B., Radosavljevic I., Tasic-Uros D., Markovic N. (2025). Nrf2 as a Molecular Guardian of Redox Balance and Barrier Integrity in IBD. Antioxidants.

[95] Wuputra K., Hsu W.H., Ku C.C., Yang Y.H., Kuo K.K., Yu F.J., Yu H.S., Nagata K., Wu D.C., Kuo C.H. (2025). The AHR-NRF2-JDP2 gene battery: Ligand-induced AHR transcriptional activation. Biochem. Pharmacol..

[96] Zhang W., Ren H., Chen W., Hu B., Feng C., Li P., Shi Y., Fang J. (2025). Nicotinamide phosphoribosyltransferase in NAD(+) metabolism: Physiological and pathophysiological implications. Cell Death Discov..

[97] Platten M., Nollen E.A.A., Rohrig U.F., Fallarino F., Opitz C.A. (2019). Tryptophan metabolism as a common therapeutic target in cancer, neurodegeneration and beyond. Nat. Rev. Drug Discov..

[98] Hou Y., Li J., Ying S. (2023). Tryptophan Metabolism and Gut Microbiota: A Novel Regulatory Axis Integrating the Microbiome, Immunity, and Cancer. Metabolites.

[99] Krautkramer K.A., Fan J., Backhed F. (2021). Gut microbial metabolites as multi-kingdom intermediates. Nat. Rev. Microbiol..

[100] Lu Z., Zhang C., Zhang J., Su W., Wang G., Wang Z. (2025). The Kynurenine Pathway and Indole Pathway in Tryptophan Metabolism Influence Tumor Progression. Cancer Med..

[101] Mezrich J.D., Fechner J.H., Zhang X., Johnson B.P., Burlingham W.J., Bradfield C.A. (2010). An interaction between kynurenine and the aryl hydrocarbon receptor can generate regulatory T cells. J. Immunol..

[102] Guarente L. (2014). Linking DNA damage, NAD(+)/SIRT1, and aging. Cell Metab..

[103] Imai S., Guarente L. (2014). NAD+ and sirtuins in aging and disease. Trends Cell Biol..

[104] Mor A., Tankiewicz-Kwedlo A., Ciwun M., Lewkowicz J., Pawlak D. (2024). Kynurenines as a Novel Target for the Treatment of Inflammatory Disorders. Cells.

[105] Diao X., Shang Q., Guo M., Huang Y., Zhang M., Chen X., Liang Y., Sun X., Zhou F., Zhuang J. (2025). Structural basis for the ligand-dependent activation of heterodimeric AHR-ARNT complex. Nat. Commun..

[106] Jones R.M., Desai C., Darby T.M., Luo L., Wolfarth A.A., Scharer C.D., Ardita C.S., Reedy A.R., Keebaugh E.S., Neish A.S. (2015). Lactobacilli Modulate Epithelial Cytoprotection through the Nrf2 Pathway. Cell Rep..

[107] Singh R., Chandrashekharappa S., Bodduluri S.R., Baby B.V., Hegde B., Kotla N.G., Hiwale A.A., Saiyed T., Patel P., Vijay-Kumar M. (2019). Enhancement of the gut barrier integrity by a microbial metabolite through the Nrf2 pathway. Nat. Commun..

[108] Nicholson J.K., Holmes E., Kinross J., Burcelin R., Gibson G., Jia W., Pettersson S. (2012). Host-gut microbiota metabolic interactions. Science.

[109] Cardona F., Andres-Lacueva C., Tulipani S., Tinahones F.J., Queipo-Ortuno M.I. (2013). Benefits of polyphenols on gut microbiota and implications in human health. J. Nutr. Biochem..

[110] Selma M.V., Espin J.C., Tomas-Barberan F.A. (2009). Interaction between phenolics and gut microbiota: Role in human health. J. Agric. Food Chem..

[111] D’Archivio M., Filesi C., Vari R., Scazzocchio B., Masella R. (2010). Bioavailability of the polyphenols: Status and controversies. Int. J. Mol. Sci..

[112] Manach C., Williamson G., Morand C., Scalbert A., Remesy C. (2005). Bioavailability and bioefficacy of polyphenols in humans. I. Review of 97 bioavailability studies. Am. J. Clin. Nutr..

[113] Ryu D., Mouchiroud L., Andreux P.A., Katsyuba E., Moullan N., Nicolet-Dit-Felix A.A., Williams E.G., Jha P., Lo Sasso G., Huzard D. (2016). Urolithin A induces mitophagy and prolongs lifespan in C. elegans and increases muscle function in rodents. Nat. Med..

[114] Andreux P.A., Blanco-Bose W., Ryu D., Burdet F., Ibberson M., Aebischer P., Auwerx J., Singh A., Rinsch C. (2019). The mitophagy activator urolithin A is safe and induces a molecular signature of improved mitochondrial and cellular health in humans. Nat. Metab..

[115] Delage B., Dashwood R.H. (2008). Dietary manipulation of histone structure and function. Annu. Rev. Nutr..

[116] Hardy T.M., Tollefsbol T.O. (2011). Epigenetic diet: Impact on the epigenome and cancer. Epigenomics.

[117] Carbonero F., Benefiel A.C., Alizadeh-Ghamsari A.H., Gaskins H.R. (2012). Microbial pathways in colonic sulfur metabolism and links with health and disease. Front. Physiol..

[118] Wallace M., Green C.R., Roberts L.S., Lee Y.M., McCarville J.L., Sanchez-Gurmaches J., Meurs N., Gengatharan J.M., Hover J.D., Phillips S.A. (2018). Enzyme promiscuity drives branched-chain fatty acid synthesis in adipose tissues. Nat. Chem. Biol..

[119] Kimura H. (2015). Signaling of hydrogen sulfide and polysulfides. Antioxid. Redox Signal..

[120] Wang M., Tang J., Zhang S., Pang K., Zhao Y., Liu N., Huang J., Kang J., Dong S., Li H. (2023). Exogenous H(2)S initiating Nrf2/GPx4/GSH pathway through promoting Syvn1-Keap1 interaction in diabetic hearts. Cell Death Discov..

[121] Mustafa A.K., Gadalla M.M., Sen N., Kim S., Mu W., Gazi S.K., Barrow R.K., Yang G., Wang R., Snyder S.H. (2009). H2S signals through protein S-sulfhydration. Sci. Signal..

[122] Soni P., Paswan S., Paul B.D., Thomas B. (2025). Intersection of H(2)S and Nrf2 signaling: Therapeutic opportunities for neurodegenerative diseases. Neurotherapeutics.

[123] Jiang J., Chan A., Ali S., Saha A., Haushalter K.J., Lam W.L., Glasheen M., Parker J., Brenner M., Mahon S.B. (2016). Hydrogen Sulfide--Mechanisms of Toxicity and Development of an Antidote. Sci. Rep..

[124] Dennis E.A., Norris P.C. (2015). Eicosanoid storm in infection and inflammation. Nat. Rev. Immunol..

[125] Serhan C.N. (2014). Pro-resolving lipid mediators are leads for resolution physiology. Nature.

[126] Basil M.C., Levy B.D. (2016). Specialized pro-resolving mediators: Endogenous regulators of infection and inflammation. Nat. Rev. Immunol..

[127] Bochkov V.N., Oskolkova O.V., Birukov K.G., Levonen A.L., Binder C.J., Stockl J. (2010). Generation and biological activities of oxidized phospholipids. Antioxid. Redox Signal..

[128] Garcia-Gimenez J.L., Ibanez-Cabellos J.S., Seco-Cervera M., Pallardo F.V. (2014). Glutathione and cellular redox control in epigenetic regulation. Free Radic. Biol. Med..

[129] Cyr A.R., Domann F.E. (2011). The redox basis of epigenetic modifications: From mechanisms to functional consequences. Antioxid. Redox Signal..

[130] Rahman I., Marwick J., Kirkham P. (2004). Redox modulation of chromatin remodeling: Impact on histone acetylation and deacetylation, NF-kappaB and pro-inflammatory gene expression. Biochem. Pharmacol..

[131] Doyle K., Fitzpatrick F.A. (2010). Redox signaling, alkylation (carbonylation) of conserved cysteines inactivates class I histone deacetylases 1, 2, and 3 and antagonizes their transcriptional repressor function. J. Biol. Chem..

[132] Niu Y., DesMarais T.L., Tong Z., Yao Y., Costa M. (2015). Oxidative stress alters global histone modification and DNA methylation. Free Radic. Biol. Med..

[133] Zhang X., Zhang Y., Wang C., Wang X. (2023). TET (Ten-eleven translocation) family proteins: Structure, biological functions and applications. Signal Transduct. Target. Ther..

[134] Li D., Guo B., Wu H., Tan L., Lu Q. (2015). TET Family of Dioxygenases: Crucial Roles and Underlying Mechanisms. Cytogenet. Genome Res..

[135] Joshi K., Liu S., Breslin S.J.P., Zhang J. (2022). Mechanisms that regulate the activities of TET proteins. Cell Mol. Life Sci..

[136] Lamadema N., Burr S., Brewer A.C. (2019). Dynamic regulation of epigenetic demethylation by oxygen availability and cellular redox. Free Radic. Biol. Med..

[137] Polytarchou C., Pfau R., Hatziapostolou M., Tsichlis P.N. (2008). The JmjC domain histone demethylase Ndy1 regulates redox homeostasis and protects cells from oxidative stress. Mol. Cell. Biol..

[138] Garcia-Gimenez J.L., Canovas-Cervera I., Nacher-Sendra E., Dolz-Andres E., Sanchez-Bernabeu A., Agundez A.B., Hernandez-Gil J., Mena-Molla S., Pallardo F.V. (2025). Oxidative stress and central metabolism pathways impact epigenetic modulation in inflammation and immune response. Free Radic. Biol. Med..

[139] Lio C.J., Yue X., Lopez-Moyado I.F., Tahiliani M., Aravind L., Rao A. (2020). TET methylcytosine oxidases: New insights from a decade of research. J. Biosci..

[140] Hancock R.L., Dunne K., Walport L.J., Flashman E., Kawamura A. (2015). Epigenetic regulation by histone demethylases in hypoxia. Epigenomics.

[141] Batie M., Rocha S. (2019). JmjC histone demethylases act as chromatin oxygen sensors. Mol. Cell. Oncol..

[142] van der Hee B., Wells J.M. (2021). Microbial Regulation of Host Physiology by Short-chain Fatty Acids. Trends Microbiol..

[143] Nitsch S., Zorro Shahidian L., Schneider R. (2021). Histone acylations and chromatin dynamics: Concepts, challenges, and links to metabolism. EMBO Rep..

[144] Zhang D., Tang Z., Huang H., Zhou G., Cui C., Weng Y., Liu W., Kim S., Lee S., Perez-Neut M. (2019). Metabolic regulation of gene expression by histone lactylation. Nature.

[145] Krautkramer K.A., Kreznar J.H., Romano K.A., Vivas E.I., Barrett-Wilt G.A., Rabaglia M.E., Keller M.P., Attie A.D., Rey F.E., Denu J.M. (2016). Diet-Microbiota Interactions Mediate Global Epigenetic Programming in Multiple Host Tissues. Mol. Cell.

[146] Krautkramer K.A., Dhillon R.S., Denu J.M., Carey H.V. (2017). Metabolic programming of the epigenome: Host and gut microbial metabolite interactions with host chromatin. Transl. Res..

[147] Takiishi T., Fenero C.I.M., Camara N.O.S. (2017). Intestinal barrier and gut microbiota: Shaping our immune responses throughout life. Tissue Barriers.

[148] Mostafavi Abdolmaleky H., Zhou J.R. (2024). Gut Microbiota Dysbiosis, Oxidative Stress, Inflammation, and Epigenetic Alterations in Metabolic Diseases. Antioxidants.

[149] Miro-Blanch J., Yanes O. (2019). Epigenetic Regulation at the Interplay Between Gut Microbiota and Host Metabolism. Front. Genet..

[150] Krautkramer K.A., Rey F.E., Denu J.M. (2017). Chemical signaling between gut microbiota and host chromatin: What is your gut really saying?. J. Biol. Chem..

[151] Domann F.E., Hitchler M.J. (2021). Aberrant redox biology and epigenetic reprogramming: Co-conspirators across multiple human diseases. Free Radic. Biol. Med..

[152] Lin M.Y., de Zoete M.R., van Putten J.P., Strijbis K. (2015). Redirection of Epithelial Immune Responses by Short-Chain Fatty Acids through Inhibition of Histone Deacetylases. Front. Immunol..

[153] Brandl N., Seitz R., Sendtner N., Muller M., Gulow K. (2025). Living on the Edge: ROS Homeostasis in Cancer Cells and Its Potential as a Therapeutic Target. Antioxidants.

[154] Garcia-Gimenez J.L., Olaso G., Hake S.B., Bonisch C., Wiedemann S.M., Markovic J., Dasi F., Gimeno A., Perez-Quilis C., Palacios O. (2013). Histone h3 glutathionylation in proliferating mammalian cells destabilizes nucleosomal structure. Antioxid. Redox Signal..

[155] Zhou M., Gao H., Wang X., Shi Z., Yang X., Li Y., Li X., Zhao Y. (2026). Nrf2 as a redox checkpoint in autoimmune joint inflammation: Microenvironmental redox control across the arthritis spectrum. Front. Immunol..

[156] Wu S., Liao X., Zhu Z., Huang R., Chen M., Huang A., Zhang J., Wu Q., Wang J., Ding Y. (2022). Antioxidant and anti-inflammation effects of dietary phytochemicals: The Nrf2/NF-kappaB signalling pathway and upstream factors of Nrf2. Phytochemistry.

[157] Casper E. (2023). The crosstalk between Nrf2 and NF-kappaB pathways in coronary artery disease: Can it be regulated by SIRT6?. Life Sci..

[158] Guo X., Hong S., He H., Zeng Y., Chen Y., Mo X., Li J., Li L., Steinmetz R., Liu Q. (2020). NFkappaB promotes oxidative stress-induced necrosis and ischemia/reperfusion injury by inhibiting Nrf2-ARE pathway. Free Radic. Biol. Med..

[159] Huchzermeier R., van der Vorst E.P.C. (2025). Aryl hydrocarbon receptor (AHR) and nuclear factor erythroid-derived 2-like 2 (NRF2): An important crosstalk in the gut-liver axis. Biochem. Pharmacol..

[160] Yu K., Li Q., Sun X., Peng X., Tang Q., Chu H., Zhou L., Wang B., Zhou Z., Deng X. (2023). Bacterial indole-3-lactic acid affects epithelium-macrophage crosstalk to regulate intestinal homeostasis. Proc. Natl. Acad. Sci. USA.

[161] Wang A., Guan C., Wang T., Mu G., Tuo Y. (2024). Lactiplantibacillus plantarum-Derived Indole-3-lactic Acid Ameliorates Intestinal Barrier Integrity through the AhR/Nrf2/NF-kappaB Axis. J. Agric. Food Chem..

[162] Peng K., Xiao S., Xia S., Li C., Yu H., Yu Q. (2024). Butyrate Inhibits the HDAC8/NF-kappaB Pathway to Enhance Slc26a3 Expression and Improve the Intestinal Epithelial Barrier to Relieve Colitis. J. Agric. Food Chem..

[163] Parada-Venegas D., De la Fuente Lopez M., Dubois-Camacho K., Landskron G., Blokzijl T., Molina H., Casanova M.C., Cui Y., Liu M., Da Costa De Pina A.M. (2025). Butyrate suppresses mucosal inflammation in inflammatory bowel disease primarily through HDAC3 inhibition in monocytes and macrophages. FEBS J..

[164] Chang P.V., Hao L., Offermanns S., Medzhitov R. (2014). The microbial metabolite butyrate regulates intestinal macrophage function via histone deacetylase inhibition. Proc. Natl. Acad. Sci. USA.

[165] Lo R., Matthews J. (2013). The aryl hydrocarbon receptor and estrogen receptor alpha differentially modulate nuclear factor erythroid-2-related factor 2 transactivation in MCF-7 breast cancer cells. Toxicol. Appl. Pharmacol..

[166] Yasuda T., Takagi T., Asaeda K., Hashimoto H., Kajiwara M., Azuma Y., Kitae H., Hirai Y., Mizushima K., Doi T. (2024). Urolithin A-mediated augmentation of intestinal barrier function through elevated secretory mucin synthesis. Sci. Rep..

[167] Krankel N. (2024). Metabolites regulating chromatin accessibility: A piece of the puzzle. Eur. Heart J..

[168] Haws S.A., Leech C.M., Denu J.M. (2020). Metabolism and the Epigenome: A Dynamic Relationship. Trends Biochem. Sci..

[169] Jiang Z., He L., Li D., Zhuo L., Chen L., Shi R.Q., Luo J., Feng Y., Liang Y., Li D. (2025). Human gut microbial aromatic amino acid and related metabolites prevent obesity through intestinal immune control. Nat. Metab..

[170] Bautista J., Echeverria C.E., Maldonado-Noboa I., Ojeda-Mosquera S., Hidalgo-Tinoco C., Lopez-Cortes A. (2025). The human microbiome in clinical translation: From bench to bedside. Front. Microbiol..

[171] Essex M., Millet Pascual-Leone B., Lober U., Kuhring M., Zhang B., Bruning U., Fritsche-Guenther R., Krzanowski M., Fiocca Vernengo F., Brumhard S. (2024). Gut microbiota dysbiosis is associated with altered tryptophan metabolism and dysregulated inflammatory response in COVID-19. npj Biofilms Microbiomes.

[172] Sinha S.R., Haileselassie Y., Nguyen L.P., Tropini C., Wang M., Becker L.S., Sim D., Jarr K., Spear E.T., Singh G. (2020). Dysbiosis-Induced Secondary Bile Acid Deficiency Promotes Intestinal Inflammation. Cell Host Microbe.

[173] Iqbal M., Yu Q., Tang J., Xiang J. (2025). Unraveling the gut microbiota’s role in obesity: Key metabolites, microbial species, and therapeutic insights. J. Bacteriol..

[174] Chulenbayeva L., Jarmukhanov Z., Kaliyekova K., Kozhakhmetov S., Kushugulova A. (2025). Quantitative Alterations in Short-Chain Fatty Acids in Inflammatory Bowel Disease: A Systematic Review and Meta-Analysis. Biomolecules.

[175] Niekamp P., Kim C.H. (2023). Microbial Metabolite Dysbiosis and Colorectal Cancer. Gut Liver.

[176] Duizer C., de Zoete M.R. (2023). The Role of Microbiota-Derived Metabolites in Colorectal Cancer. Int. J. Mol. Sci..

[177] Zarei P., Sedeh P.A., Vaez A., Keshteli A.H. (2025). Using metabolomics to investigate the relationship between the metabolomic profile of the intestinal microbiota derivatives and mental disorders in inflammatory bowel diseases: A narrative review. Res. Pharm. Sci..

[178] Zhang Y., Thomas J.P., Korcsmaros T., Gul L. (2024). Integrating multi-omics to unravel host-microbiome interactions in inflammatory bowel disease. Cell Rep. Med..

[179] Wang Q., Ye J., Fang D., Lv L., Wu W., Shi D., Li Y., Yang L., Bian X., Wu J. (2020). Multi-omic profiling reveals associations between the gut mucosal microbiome, the metabolome, and host DNA methylation associated gene expression in patients with colorectal cancer. BMC Microbiol..

[180] Onwuka S., Bravo-Merodio L., Gkoutos G.V., Acharjee A. (2024). Explainable AI-prioritized plasma and fecal metabolites in inflammatory bowel disease and their dietary associations. iScience.

[181] Kalla R., Adams A.T., Nowak J.K., Bergemalm D., Vatn S., Ventham N.T., Kennedy N.A., Ricanek P., Lindstrom J., Consortium I.B.-C. (2023). Analysis of Systemic Epigenetic Alterations in Inflammatory Bowel Disease: Defining Geographical, Genetic and Immune-Inflammatory influences on the Circulating Methylome. J. Crohn’s Colitis.

[182] Chen W., Wang D., Deng X., Zhang H., Dong D., Su T., Lu Q., Jiang C., Ni Q., Cui Y. (2024). Bile acid profiling as an effective biomarker for staging in pediatric inflammatory bowel disease. Gut Microbes.

[183] Vich Vila A., Zhang J., Liu M., Faber K.N., Weersma R.K. (2024). Untargeted faecal metabolomics for the discovery of biomarkers and treatment targets for inflammatory bowel diseases. Gut.

[184] Vich Vila A., Hu S., Andreu-Sanchez S., Collij V., Jansen B.H., Augustijn H.E., Bolte L.A., Ruigrok R., Abu-Ali G., Giallourakis C. (2023). Faecal metabolome and its determinants in inflammatory bowel disease. Gut.

[185] Liu M., Guo S., Wang L. (2024). Systematic review of metabolomic alterations in ulcerative colitis: Unveiling key metabolic signatures and pathways. Ther. Adv. Gastroenterol..

[186] Mestrovic A., Perkovic N., Bozic D., Kumric M., Vilovic M., Bozic J. (2024). Precision Medicine in Inflammatory Bowel Disease: A Spotlight on Emerging Molecular Biomarkers. Biomedicines.

[187] Paramsothy S., Kamm M.A., Kaakoush N.O., Walsh A.J., van den Bogaerde J., Samuel D., Leong R.W.L., Connor S., Ng W., Paramsothy R. (2017). Multidonor intensive faecal microbiota transplantation for active ulcerative colitis: A randomised placebo-controlled trial. Lancet.

[188] Haifer C., Paramsothy S., Kaakoush N.O., Saikal A., Ghaly S., Yang T., Luu L.D.W., Borody T.J., Leong R.W. (2022). Lyophilised oral faecal microbiota transplantation for ulcerative colitis (LOTUS): A randomised, double-blind, placebo-controlled trial. Lancet Gastroenterol. Hepatol..

[189] Costello S.P., Hughes P.A., Waters O., Bryant R.V., Vincent A.D., Blatchford P., Katsikeros R., Makanyanga J., Campaniello M.A., Mavrangelos C. (2019). Effect of Fecal Microbiota Transplantation on 8-Week Remission in Patients with Ulcerative Colitis: A Randomized Clinical Trial. JAMA.

[190] Pietrzak A., Banasiuk M., Szczepanik M., Borys-Iwanicka A., Pytrus T., Walkowiak J., Banaszkiewicz A. (2022). Sodium Butyrate Effectiveness in Children and Adolescents with Newly Diagnosed Inflammatory Bowel Diseases-Randomized Placebo-Controlled Multicenter Trial. Nutrients.

[191] Teubner J.P., Tumen D., Kandulski A., Heumann P., Mester P., Aschenbrenner E., Pollinger K., Gunckel M., Volz B., Hein T. (2026). CRISPR-Cas9 screen reveals that inhibition of enhancer of zeste homolog 2 sensitizes malignant T cells to dimethyl-fumarate-induced cell death. FEBS J..

[192] Vernocchi P., Del Chierico F., Putignani L. (2020). Gut Microbiota Metabolism and Interaction with Food Components. Int. J. Mol. Sci..

[193] Hitch T.C.A., Hall L.J., Walsh S.K., Leventhal G.E., Slack E., de Wouters T., Walter J., Clavel T. (2022). Microbiome-based interventions to modulate gut ecology and the immune system. Mucosal Immunol..

[194] Nusbaum D.J., Sun F., Ren J., Zhu Z., Ramsy N., Pervolarakis N., Kunde S., England W., Gao B., Fiehn O. (2018). Gut microbial and metabolomic profiles after fecal microbiota transplantation in pediatric ulcerative colitis patients. FEMS Microbiol. Ecol..

[195] Wu R., Xiong R., Li Y., Chen J., Yan R. (2023). Gut microbiome, metabolome, host immunity associated with inflammatory bowel disease and intervention of fecal microbiota transplantation. J. Autoimmun..

[196] Goldis A., Dragomir R., Mercioni M.A., Sirca D., Goldis C., Enatescu I., Olariu L., Belei O. (2025). Clinical Efficacy of Sodium Butyrate in Managing Pediatric Inflammatory Bowel Disease. Life.

[197] Jamka M., Kokot M., Kaczmarek N., Bermagambetova S., Nowak J.K., Walkowiak J. (2021). The Effect of Sodium Butyrate Enemas Compared with Placebo on Disease Activity, Endoscopic Scores, and Histological and Inflammatory Parameters in Inflammatory Bowel Diseases: A Systematic Review of Randomised Controlled Trials. Complement. Med. Res..

[198] Schmitt A., Xu W., Bucher P., Grimm M., Konantz M., Horn H., Zapukhlyak M., Berning P., Brandle M., Jarboui M.A. (2021). Dimethyl fumarate induces ferroptosis and impairs NF-kappaB/STAT3 signaling in DLBCL. Blood.

[199] Nicolay J.P., Muller-Decker K., Schroeder A., Brechmann M., Mobs M., Geraud C., Assaf C., Goerdt S., Krammer P.H., Gulow K. (2016). Dimethyl fumarate restores apoptosis sensitivity and inhibits tumor growth and metastasis in CTCL by targeting NF-kappaB. Blood.

[200] Schroeder A., Warnken U., Roth D., Klika K.D., Vobis D., Barnert A., Bujupi F., Oberacker T., Schnolzer M., Nicolay J.P. (2017). Targeting Thioredoxin-1 by dimethyl fumarate induces ripoptosome-mediated cell death. Sci. Rep..

[201] Benard M.V., de Goffau M.C., Blonk J., Hugenholtz F., van Buuren J., Paramsothy S., Kaakoush N.O., D’Haens G., Borody T.J., Kamm M.A. (2025). Gut Microbiota Features in Relation to Fecal Microbiota Transplantation Outcome in Ulcerative Colitis: A Systematic Review and Meta-Analysis. Clin. Gastroenterol. Hepatol..

[202] Mousa W.K., Al Ali A. (2024). The Gut Microbiome Advances Precision Medicine and Diagnostics for Inflammatory Bowel Diseases. Int. J. Mol. Sci..

[203] Zhang X., Li L., Butcher J., Stintzi A., Figeys D. (2019). Advancing functional and translational microbiome research using meta-omics approaches. Microbiome.

[204] Jagirdhar G.S.K., Perez J.A., Perez A.B., Surani S. (2023). Integration and implementation of precision medicine in the multifaceted inflammatory bowel disease. World J. Gastroenterol..

[205] Ananthakrishnan A.N. (2024). Precision medicine in inflammatory bowel diseases. Intest. Res..

[206] Lee H.S., Cleynen I. (2019). Molecular Profiling of Inflammatory Bowel Disease: Is It Ready for Use in Clinical Decision-Making?. Cells.

[207] Chen-Liaw A., Aggarwala V., Mogno I., Haifer C., Li Z., Eggers J., Helmus D., Hart A., Wehkamp J., Lamouse-Smith E.S.N. (2025). Gut microbiota strain richness is species specific and affects engraftment. Nature.

[208] Urtecho G., Moody T., Huang Y., Sheth R.U., Richardson M., Descamps H.C., Kaufman A., Lekan O., Zhang Z., Velez-Cortes F. (2024). Spatiotemporal dynamics during niche remodeling by super-colonizing microbiota in the mammalian gut. Cell Syst..

[209] Verdu E.F., Schuppan D. (2021). Co-factors, Microbes, and Immunogenetics in Celiac Disease to Guide Novel Approaches for Diagnosis and Treatment. Gastroenterology.

